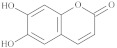# Antagonists of Vitamin K—Popular Coumarin Drugs and New Synthetic and Natural Coumarin Derivatives

**DOI:** 10.3390/molecules25061465

**Published:** 2020-03-24

**Authors:** Kinga Kasperkiewicz, Michał B. Ponczek, Jacek Owczarek, Piotr Guga, Elżbieta Budzisz

**Affiliations:** 1 Department of Cosmetic Raw Materials Chemistry, Faculty of Pharmacy, Medical University of Lodz, Muszynskiego 1, 90-151 Łódź, Poland; kinga.kasperkiewicz@umed.lodz.pl; 2 Department of General Biochemistry, Faculty of Biology and Environmental Protection, University of Lodz, Pomorska 141/143, 90-236 Łódź, Poland; michal.ponczek@biol.uni.lodz.pl; 3 Department of Medical Pharmacy, Faculty of Pharmacy, Medical University of Lodz, Muszynskiego 1, 90-151 Łódź, Poland; jacek.owczarek@umed.lodz.pl; 4 Centre of Molecular and Macromolecular Studies, Polish Academy of Sciences, Department of Bioorganic Chemistry, Sienkiewicza 112, 90-363 Łódź, Poland; pguga@cbmm.lodz.pl

**Keywords:** coumarins, coumarin derivatives, oral anticoagulants, cardiovascular diseases, vitamin K, vitamin K antagonists

## Abstract

Many natural coumarins and their chemically synthesized analogs and derivatives exert diverse properties, such as anticancer, antioxidant, anti-inflammatory, or anticoagulant, with the latter being of the utmost importance. The widely used warfarin, acenocoumarol, and phenprocoumon exert anticoagulant properties by inhibiting the vitamin K epoxide reductase complex. In this interdisciplinary review, we present biochemical principles of the coagulation processes and possible methods for their tuning based on the use of coumarins. We also summarize chemical methods of synthesis of coumarins and discuss structures and properties of those that have been used for a long time, as well as newly synthesized compounds. Brief information on the clinical use of coumarins and other anticoagulant drugs is given, including the severe effects of overdosing and methods for reversing their action.

## 1. Blood Coagulation: A Process Dependent on Vitamin K Wanted or Dreadful

**Figure 1:**
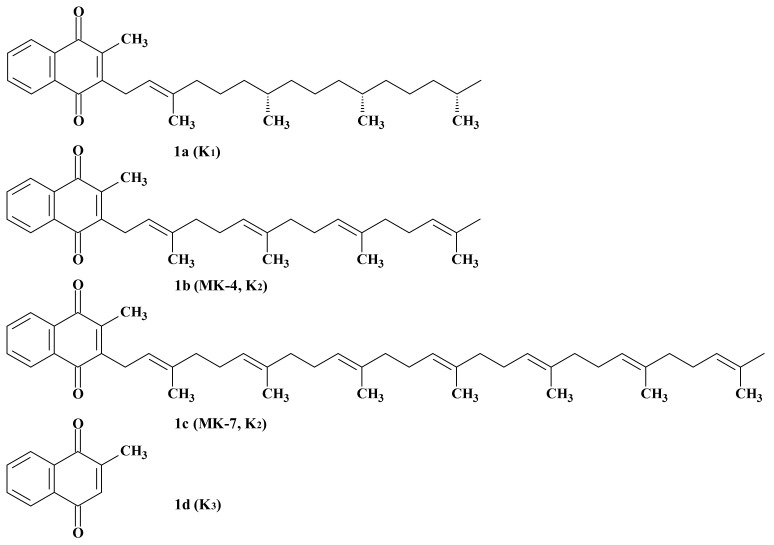
Chemical structures of vitamin K (**1**). (**1a**) K_1_; (**1b** and **1c**) K_2_; and (**1d**) K_3_.

Diseases that attack the blood circulation system in humans are the most fatal, next to cancer, on all continents except Africa. There are numerous types of these diseases, including hemophilia, angina and myocardial infarction, stroke, hypertensive heart disease, rheumatic heart disease, cardiomyopathy, heart arrhythmia, and several others. Despite their variety, the risk factors are very common in developed countries and include high blood pressure, smoking, lack of exercise, diabetes, obesity, poor diet, high blood cholesterol, and overuse of alcohol [1]; therefore, tens of millions of people worldwide suffer from cardiovascular diseases. Nonetheless, in some countries proper education and a healthier lifestyle deliver optimistic results, i.e., reduced numbers of fatalities. Some of the aforementioned diseases are related to a blood coagulation phenomenon. The blood is capable of repairing traumatic injury and stopping bleeding due to a coagulation process that leads to formation of clots. Poor, inefficient coagulation is a rare genetic disorder (called hemophilia), in which blood does not clot normally due to an insufficient level of blood-clotting proteins (clotting factors). A hemophilia patient can bleed for a long time even from a small cut, but a significant threat comes from bleeding inside the body (joint, muscle and sub-, internal, and intracranial bleedings), which can be fatal due to the damage done to organs and tissues. The main treatment for severe hemophilia involves intravenously receiving a replacement of the specific clotting factor, either to combat an ongoing bleeding episode, or to prevent bleeding episodes on a regular basis. Replacement of the clotting factor is achieved from donated blood, but recombinant clotting factors are also available. It should also be noted that the coagulation factors dependent on vitamin K (**1**) (for the vitamin K characteristics see Figure 1) are synthesized in the liver, and therefore liver-related diseases can result in lower blood levels of vitamin K-dependent clotting factors, with concomitant susceptibility for uncontrolled bleeding. 

**Figure 2:**
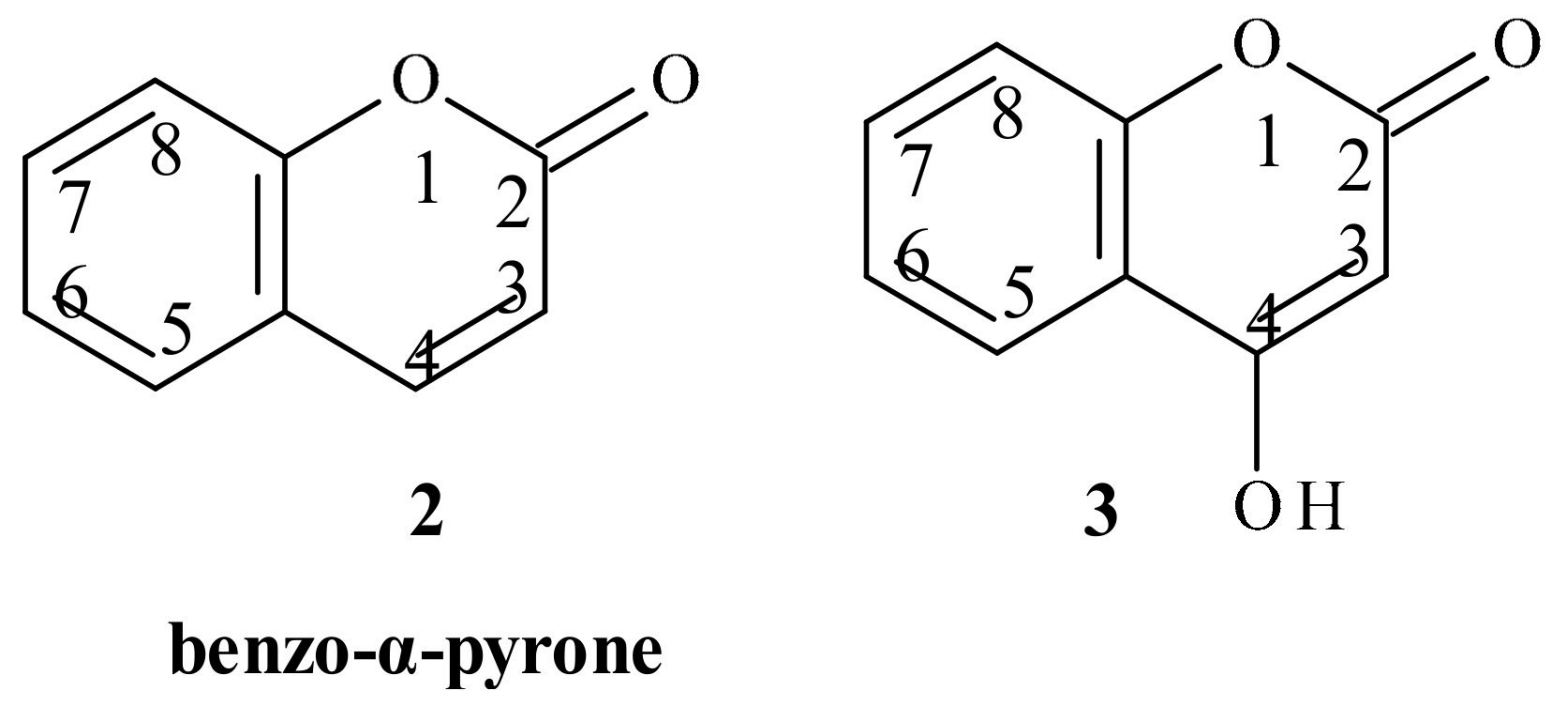
Structures of coumarin and 4-hydroxycoumarin.

The first experiments that showed decreased vitamin K activity with the use of an anticoagulant compound (so it acts as a vitamin K antagonist, VKA) were done in 1954 [2]. The mechanism of action of VKAs is based on the inhibition of a vitamin K epoxide reductase (VKOR, see Section 2 and Section 4). The VKAs interact with widely used drugs, for example, with barbiturates, carbamazepines, rifampicin, nafcillin, and dikloksacilin. Long-term smoking, alcohol abuse or certain dietary components [3] can also negatively affect the VKAs’ action [4]. Derivatives of coumarin (**2** in Figure 2) and 4-hydroxycoumarin (**3** in Figure 2) are important orally administered anticoagulants, which antagonize VKOR and prevent recycling of vitamin K.

**Figure 3:**
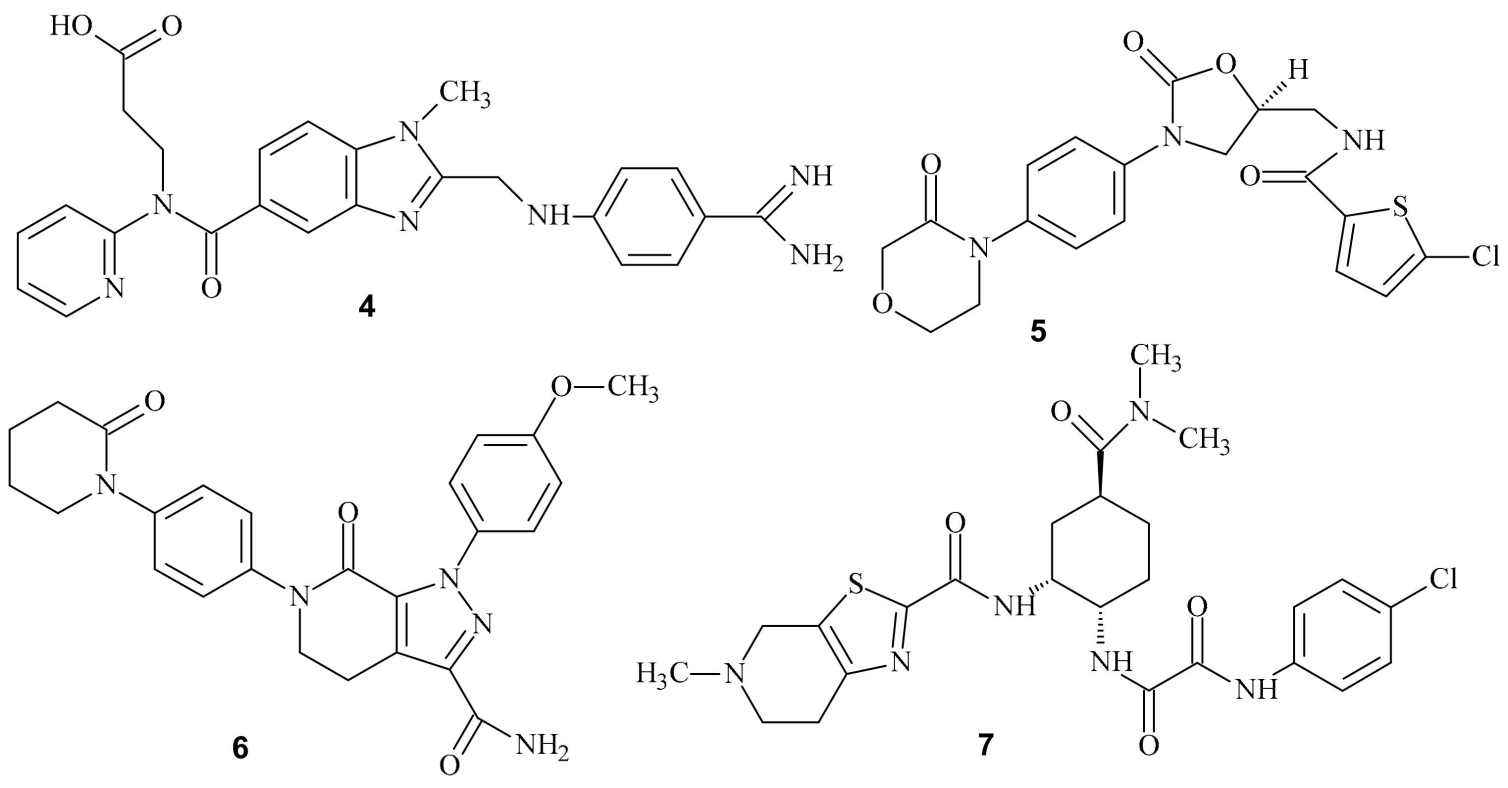
Structures of selected non-VKA oral anticoagulants (NOAC).

In contrast to hemophilia, there are diseases (in their chronic or acute forms), in which coagulation is too strong, and therefore there is too much clotting. In the most dangerous instances, acute forms (strokes) develop, which are caused by heart, brain, or blood vessel defects. In chronic, less dangerous cases, the symptoms of excessive clotting can be, to a certain extent, neutralized with anticoagulants. These drugs are also prescribed to prevent blood clots in people who have replacement heart valves, people with an irregular heartbeat, or those who had a heart attack, stroke, or a prior blood clot in the deep veins of the arms or legs. Venous thromboembolic (VTE) disease is the third, most common cardiovascular disease with an annual incidence of 100 to 200 per 100,000 inhabitants [5]. It can be fatal in its acute phase or can lead to chronic disease and disability, but it can also often be prevented [6]. Acute incidents of VTE must be treated with anticoagulants as soon as possible [7], but the drugs are also given in a long-term regime (minimum three months) to prevent the return of the disease [8,9]. For this purpose, the most frequently used drugs are VKAs, although the introduction of non-VKA oral anticoagulants (NOAC) such as dabigatran (**4** in Figure 3) which is a direct inhibitor of thrombin; and rivaroxaban (**5** in Figure 3), apixaban (**6** in Figure 3), or edoxaban (**7** in Figure 3) which are direct inhibitors of coagulation factor X, should be endorsed. The latter do not require frequent monitoring of blood coagulation parameters but can still cause bleeding and often require assessment of kidney function, especially in patients with kidney problems [10,11,12,13].

There is also a much related and equally dangerous disease which is pulmonary embolism (PE), and in patients with its high and medium clinical probability, an immediate parenteral anticoagulation should be initiated [14]. In addition, patients with atrial fibrillation (AF), who have one high risk factor or two or more moderate risk factors for stroke, must be considered for chronic anticoagulation. In 2010, the number of people with AF worldwide was estimated at 20.9 million men and 12.6 million women [15]. In many cases, AF becomes fatal due to cerebral stroke, the risk of which can be significantly reduced by an anticoagulation treatment. The chronic treatment with VKAs is recommended for patients after implantation of artificial heart valves and with moderate or severe mitral valve stenosis [16,17,18]. Currently, developed NOACs should not be administered to this group of patients. In addition, pregnancy excludes NOACs, whereas VKAs can be used during the second and third trimester (until the 36th week) in women needing a low dose of the drugs (warfarin < 5 mg/day, see Section 5.1 and acenocoumarol < 2 mg/day) [19].

The risk of VKA-related major bleeding increases significantly if the international normalized ratio (INR) of the prothrombin time exceeds 4.5 and boosts exponentially above 6.0. Clinical problems in maintaining INR during treatment result from narrow therapeutic indices of these drugs, i.e., a situation where small differences in dose or concentration in the blood can lead to dose and blood concentration dependency, serious therapeutic failures, or adverse drug reactions. Thus, for a large number of patients, either ineffectiveness of therapy or increased risk of bleeding are noted, the latter mainly from the gastrointestinal tract [20,21,22]. Intracranial and intraarticular bleeding, epistaxis, and hematoma are also often observed [20]. For warfarin overdose related bleeding, vitamin K is a reversal agent, although it does not bring rapid effects, because it only facilitates synthesis of vitamin K-dependent clotting factors. To reverse dabigatran (a nonvitamin K anticoagulant), a monoclonal antibody fragment is used, whereas recombinant modified human activated factor X helps to neutralize the side effects of apixaban and rivaroxaban. It should be noted, that NOACs therapeutic indices are much improved as compared with VKAs because they do not require permanent dose control and interact less with other drugs and food. However, important contraindications for NOACs, i.e., renal failure, artificial heart valves, moderate or severe mitral valve stenosis, pregnancy, and therapy costs, force the use of VKAs, mostly warfarin or acenocoumarol [23].

Whereas there are four main groups of drugs that inhibit blood clotting, i.e., vitamin K antagonists, indirect and direct inhibitors of thrombin activity, and direct inhibitors of the clotting factor Xa activity, this review is focused on the anticoagulation properties of several natural coumarins (isolated mostly from plant extracts) and their chemically synthesized analogs, which act as vitamin K antagonists.

## 2. Coagulation Pathways

The function of the coagulation processes is to keep hemostasis, which is blockage of bleeding, either internal or external. This is a very complicated phenomenon which takes place with the participation of many proteins. Primary hemostasis is an aggregation of platelets that form a plug at the surface of exposed endothelial cells. This process is supported by secondary hemostasis (coagulation cascade), where the platelet plug is stabilized with a fibrin lattice.

The coagulation cascade process includes two separate pathways, extrinsic and intrinsic, which converge at a specific point, leading to fibrin activation. The intrinsic pathway is activated by damage inside the vascular system, and is triggered by platelets, exposed endothelium (the inner cellular lining of blood vessels and lymphatics), chemicals, or collagen. This pathway develops with the participation of several proteins, i.e., factors I, II, IX, X, XI, and XII, namely fibrinogen, prothrombin, Christmas factor, Stuart–Prower factor, plasma thromboplastin, and Hageman factor, respectively. It begins with the activation of factor XII (a zymogen, inactivated serine protease), which upon contact with endothelial collagen (exposed when the endothelium gets damaged) converts into factor XIIa (activated serine protease). Within this cascade, factor XIIa acts as a catalyst to activate factor XI to factor XIa, which then activates factor IX to factor IXa, at the end of the sequence which allowing conversion of factor X into factor Xa. The extrinsic pathway consists of factors I, II, VII, and X. Once the blood vessel gets damaged, the endothelial cells release a tissue factor, which activates factor VII (called stable factor, or proconvertin) to factor VIIa, and the latter activates factor X into factor Xa. At this point, the extrinsic and intrinsic pathways merge and factor Xa and factor Va activate factor II to IIa (prothrombin to thrombin), which eventually converts fibrinogen to fibrin. The fibrin subunits aggregate to form fibrin strands, and factor XIII uses them to form the aforementioned fibrin lattice.

Various substances are required for the proper functioning of the coagulation cascade. In a certain step, the intermediate complexes of factors Xa and IXa utilize the terminal γ-carboxyl groups (introduced at the side chain of glutamate residues) to interact with the phospholipid surfaces expressed by platelets, and this interaction is necessary for the clot formation to commence. The immature clotting factors are decorated with these additional γ-carboxyl groups upon action of hepatic γ-glutamylcarboxylase (GGCX), for which vitamin K is an essential factor. In this process, vitamin K gets oxidized to form vitamin K epoxide, which is reduced back to its active form with vitamin K epoxide reductase (vitamin K oxide reductase, VKOR, EC 1.17.4.4). Thus, VKOR is a pharmacologically important target for anticoagulant drugs, because if the enzyme gets inhibited, increasing deficiency of the active vitamin K prevents blood clotting. For that purpose, over the years, the most popular have been and still are two drugs, i.e., heparin (polysaccharide of 3000 to 30,000 Da molecular weight, delivered by injection) and warfarin (Coumadin, a coumarin derivative, administered orally). These drugs, although effective, also often cause severe side effects, including critical bleedings, however, there are several reversal agents available for the patients suffering from taking warfarin. On the one hand, the low platelet levels caused by heparin use can cause new clots in the blood vessels and this can lead to a stroke or heart attack. On the other hand, warfarin relatively easily makes the blood “too thin” and this can increase the risk of brain hemorrhage, a type of stroke caused by bleeding in the brain. In addition to those devastating effects, the use of anticoagulants can result in many less harsh side effects such as passing blood in the urine, severe bruising, prolonged nosebleeds, bleeding gums, or heavy periods in women. Speaking a bit in advance, recently developed oral anticoagulants (called “blood thinners”), i.e., dabigatran (Pradaxa), rivaroxaban (Xarelto), and apixaban (Eliquis), surpass heparin and warfarin in reduced risk of stroke, but also can cause bleeding. Thus, there is an ongoing search for new effective anticoagulants with less severe side effects.

## 3. Members of the Vitamin K Family

Vitamin K is a group of structurally similar, fat-soluble vitamins, required not only for blood coagulation but also to control the binding of calcium. Deficiency of vitamin K (**1**) can lead to osteoporosis and can result in calcification of arteries and other soft tissues. The vitamin K family (Figure 1) includes vitamin K_1_ (**1a** in Figure 1) and a set of isoprenoid 3-substituted derivatives of 2-methyl-1,4-naphthoquinone, called vitamin K_2_ (**1b** in Figure 1). Vitamin K_1_ is known as phylloquinone and is present in the highest amounts in green leafy vegetables. Bacteria in gastrointestinal microbiota can convert K_1_ into more “unsaturated” vitamin K_2_ (menaquinone), with MK-4 (**1b** in Figure 1) (menaquinone vitamin K with four unsaturated bonds in the side chain) being the most common type in animal tissues. Interestingly, due to the frequent elongation of the isoprenoid side chain, a range of vitamin K_2_ congeners (**1c** in Figure 1) (MK_n_, *n* = 1–13, K_2_ vitamers) is produced. Synthetic vitamin K_3_ (**1d** in Figure 1) (menadione) without the side chain in the 3-position cannot exert all the functions of the K vitamins. However, it is metabolized to yield menaquinones, and hence should be called a provitamin.

**Scheme 1:**
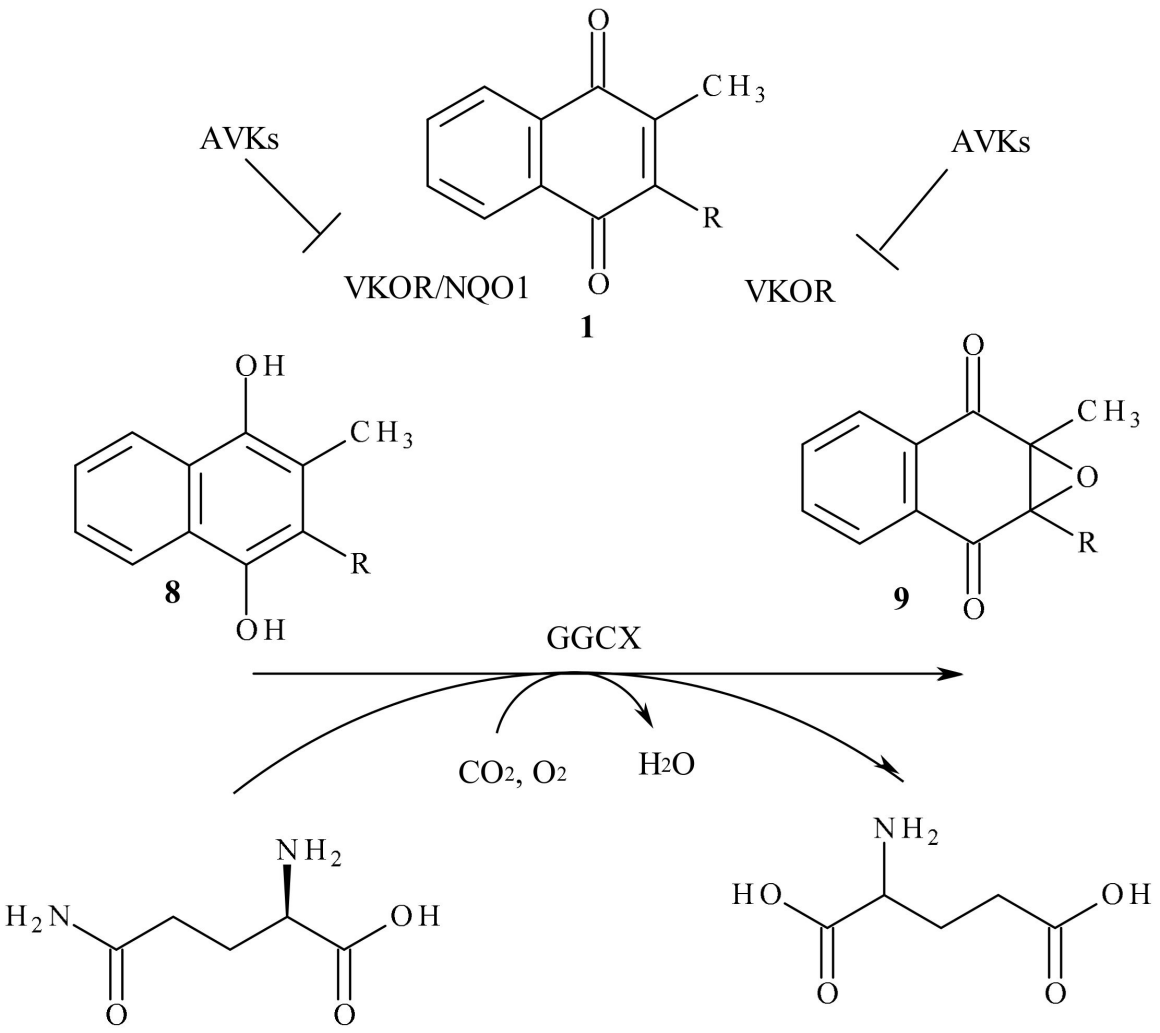
Vitamin K cycle.

Upon the entering into the coagulation process, the “idle”, 1,4-naphthoquinone form of vitamin K (**1** in Scheme 1, R represents a partially unsaturated hydrocarbon chain) is transformed to the active, hydroquinone form (**8** in Scheme 1, a reduced derivative). The reduced intermediate (**8** in Scheme 1) participates in post-translational modification of specific glutamate residues (Glu) into γ-carboxylated species (Gla), which are required for the activity of vitamin K-dependent proteins (VKDP) [24]. The coagulation factors II, VII, IX, and X, all require the Gla units for proper work and in their shortage they cannot bind Ca^2+^ ions or create the enzymatic complexes on the surface of cell membranes [25,26]. The carboxylation is performed by GGCX, which also utilizes CO_2_, and O_2_. In this process, the reduced derivative (**8** in Scheme 1) is oxidized into vitamin K 2,3-epoxide (**9** in Scheme 1) (other modified proteins, which also contain Gla residues, are anticoagulant protein C, protein S, and protein Z which selectively inactivate or facilitate inactivation of factors Va, VIIIa, or Xa). The epoxide (**9** in Scheme 1) must be reduced back to the reduced derivative (**8** in Scheme 1) before it can re-enter the path, and this conversion is executed by VKOR [27,28].

## 4. Vitamin K Oxide Reductase, VKOR

**Figure 4:**
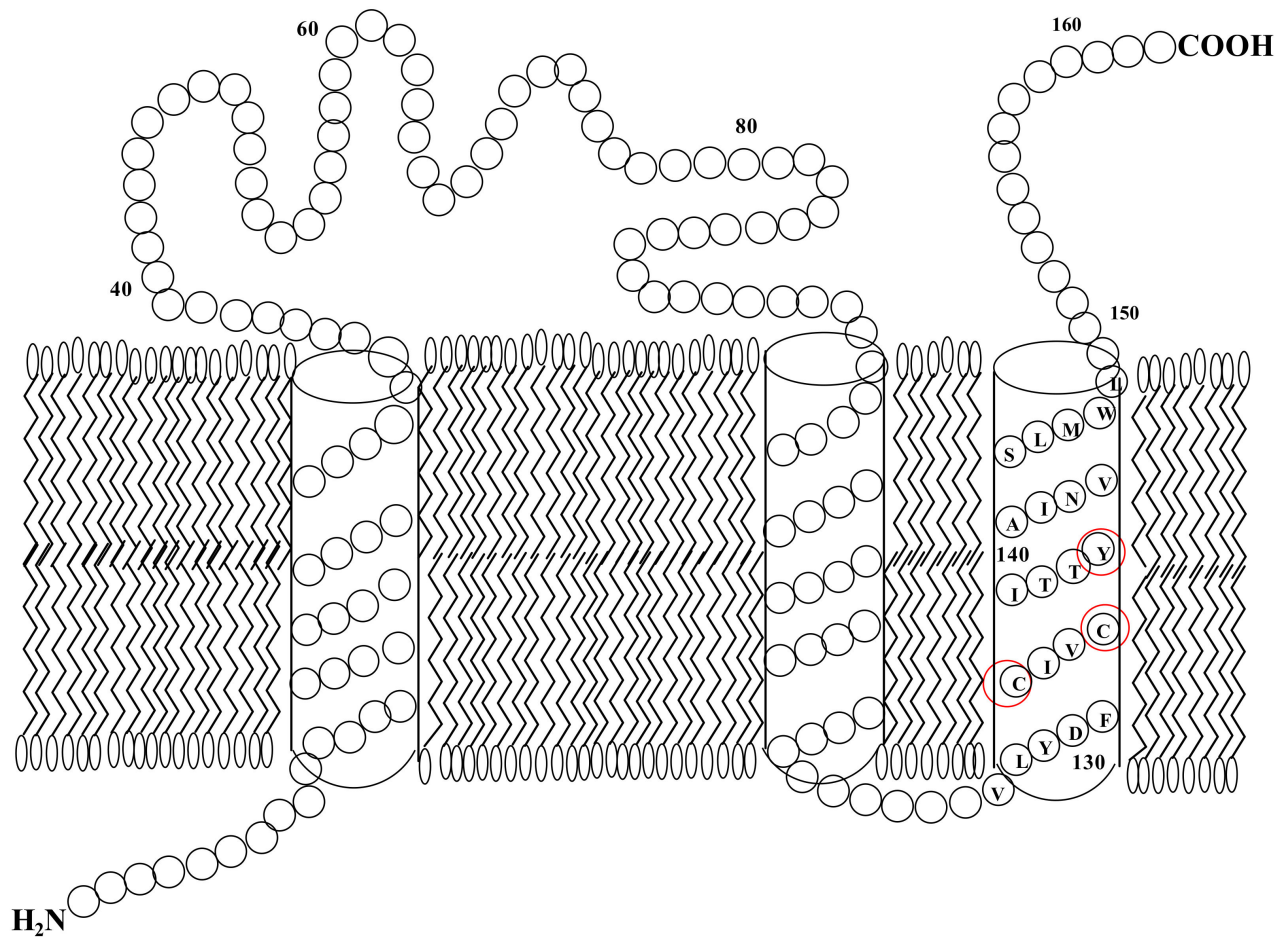
Topology of the VKOR protein, the amino terminus resides in the ER lumen and the carboxy terminus resides in the cytoplasm [30].

VKOR is an integral membrane protein which crosses the endoplasmic reticulum (ER) membrane three times (Figure 4). It has been proposed that for the enzyme to be active, the disulfide bridge (spanning cysteines 132 and 135) in the active site needs to be reduced. During each catalytic cycle, the two sulfhydryl groups are oxidized back to a disulfide bond, while two –SH hydrogen atoms and the oxygen atom (eliminated from the epoxide moiety) form a water molecule (for more detailed mechanism see [29]). VKOR is inhibited by warfarin and other coumarin derivatives. The active site and the proposed warfarin binding site (tyrosine 139) are both located in the third transmembrane helix, i.e., the first one from the C-terminus. Unfortunately, for a number of patients, coumarin-based drugs are ineffective, or their efficacy is much reduced. Table 1 shows mutations identified in VKOR being attributed to this coumarin resistance (CMRES) phenomenon.

In addition to the identification of naturally existing mutations, mutagenesis studies were performed which showed the amino acid residues vital for the VKOR activity or strength of the coumarin inhibitory effect (Table 2).

One can conclude that a better understanding of the vitamin K cycle and active complexes of VKOR is necessary to improve selectivity and efficacy of coumarin-based drugs [34,35]. These efforts must be accompanied by synthesis and testing of new members of this family.

## 5. Coumarins: Synthesis, Structures, and Properties

**Figure 5:**
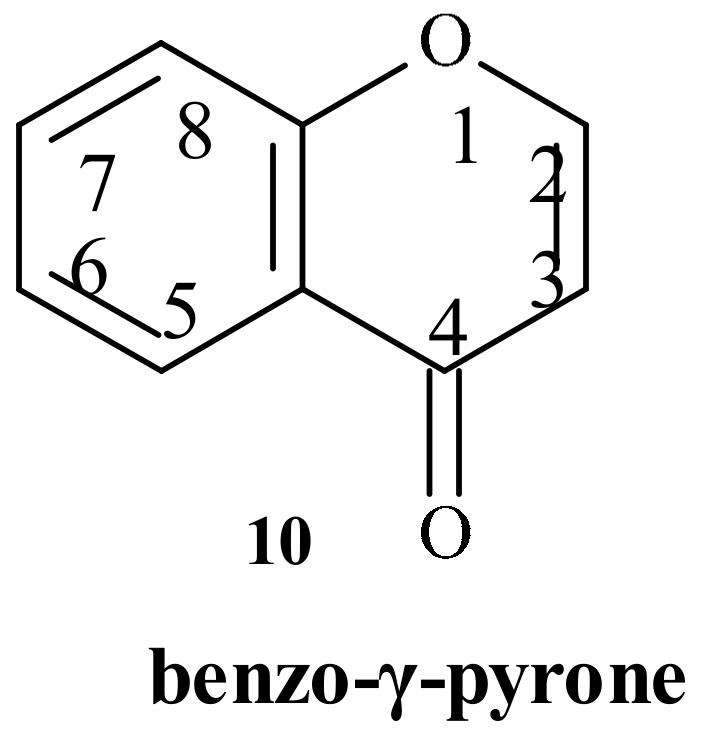
Structure of chromone.

Coumarins, i.e., derivatives of benzo-α-pyrone (coumarin, 2*H*-chromen-2-one) (**2** in Figure 2), belong to a family of benzopyrones which also contains compounds with a benzo-γ-pyrone skeleton (chromone, 4*H*-chromen-4-one) (**10** in Figure 5) [36].

**Scheme 2:**
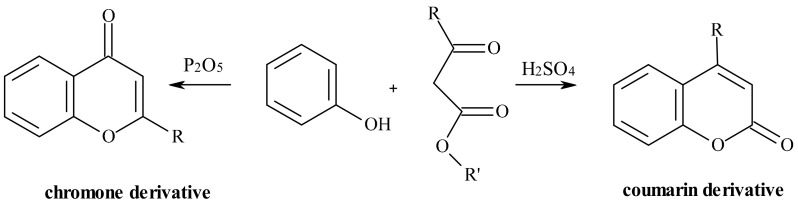
Products of cyclization according to Simonis (2-substituted chromone derivatives) and Pechmann (4-substituted coumarin derivatives).

The latter are often synthesized in so-called Simonis chromone cyclization (Scheme 2), the reaction of phenols and β-ketoesters driven by the presence of phosphorus pentoxide. Although this reaction is, to a certain extent, similar to the Pechmann synthesis of coumarins [37] (catalyzed by acids, vide infra), the different outcome results from the initial activation of the ketone moiety in the *β*-ketoester by P_2_O_5_ followed by a reaction with the phenol hydroxyl group and not with arene group.

**Figure 6:**
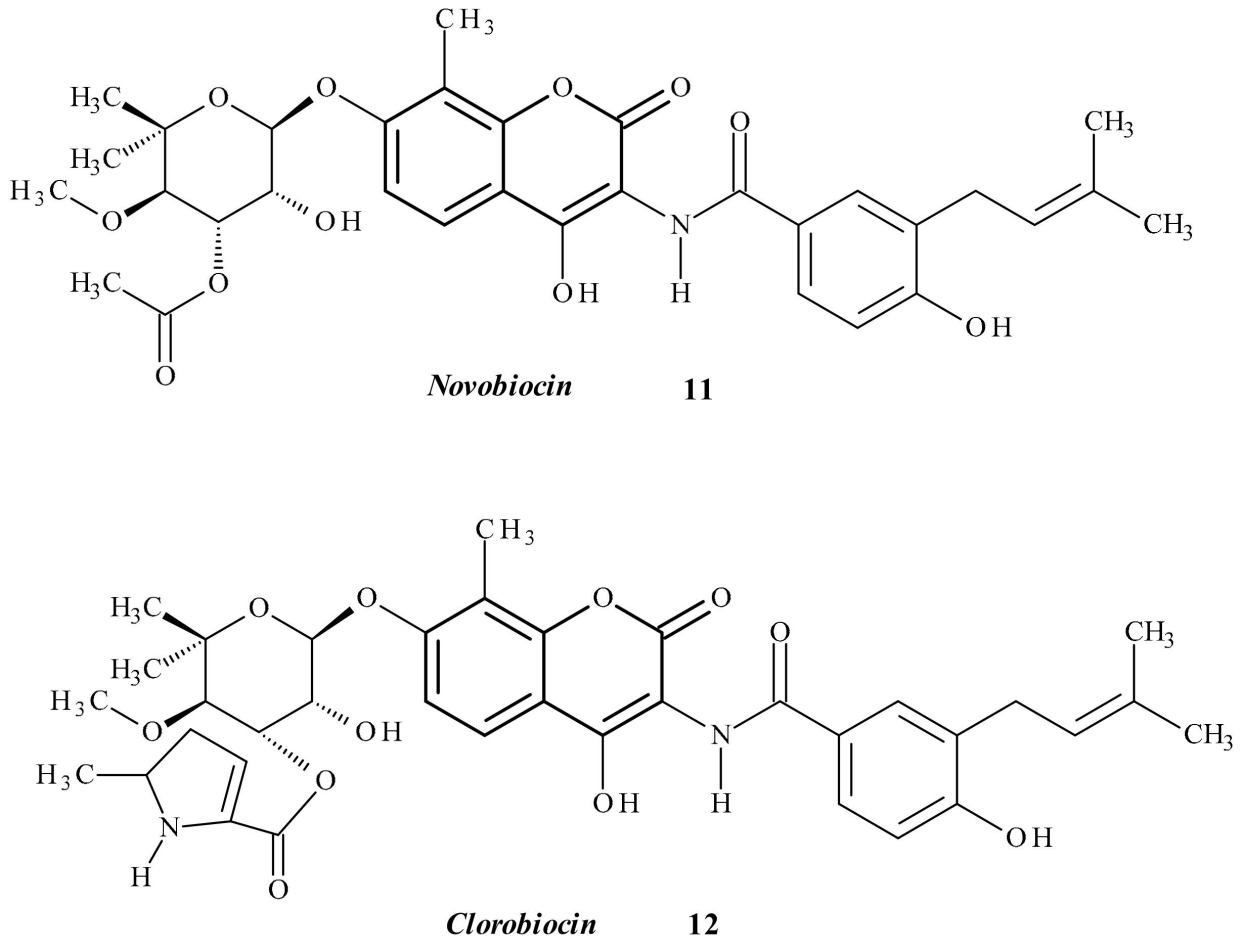
Chemical structures of novobiocin and clorobiocin.

Compounds possessing these structural motifs are not sporadic in nature [38] and have been found in citrus fruits, vegetables (tomatoes, broccoli, paprika), legumes, celeriacs, cinnamon, peppermint, green tea, lovage, papilionaceous plants, as well as in nuts and coffee. A high concentration of coumarins was detected in the essential oils cassia, cinnamon bark, and lavender oil. Coumarin was isolated in the nineteenth century from seeds of a tree *Dipteryx odorata* (commonly known as “cumaru” or “kumaru”) [39,40]. Coumarins decorated with various substituents are secondary metabolites [41] and possess numerous applications. For example, novobiocin (isolated from *Streptomyces niveus*, **11** in Figure 6) and clorobiocin (**12** in Figure 6) are natural antimicrobial compounds [42]. Aside of anticoagulant properties, different coumarins exert antiviral [43,44,45] antioxidant [46] or antidepressant [47] activity, but also inhibit acetylcholinesterase (involved in Alzheimer’s disease) [48], aromatase (a target in breast cancer therapy) [49], and squalene-hopene cyclase (cholesterol lowering and antitrypanosomal drugs) [50]. They also exert antifungal [51] and anticancer activities [52]. Certain compounds derived from 4-hydroxycoumarin (**3** in Figure 2) protect liver cells from damage by peroxides [53]. Some coumarins have found applications in perfumery [54], but also as dyes in laser technology [55], fluorescent indicators [56], nonlinear optical chromophores, fluorescent whiteners, material for optical recording, and solar energy collectors [57].

**Figure 7:**
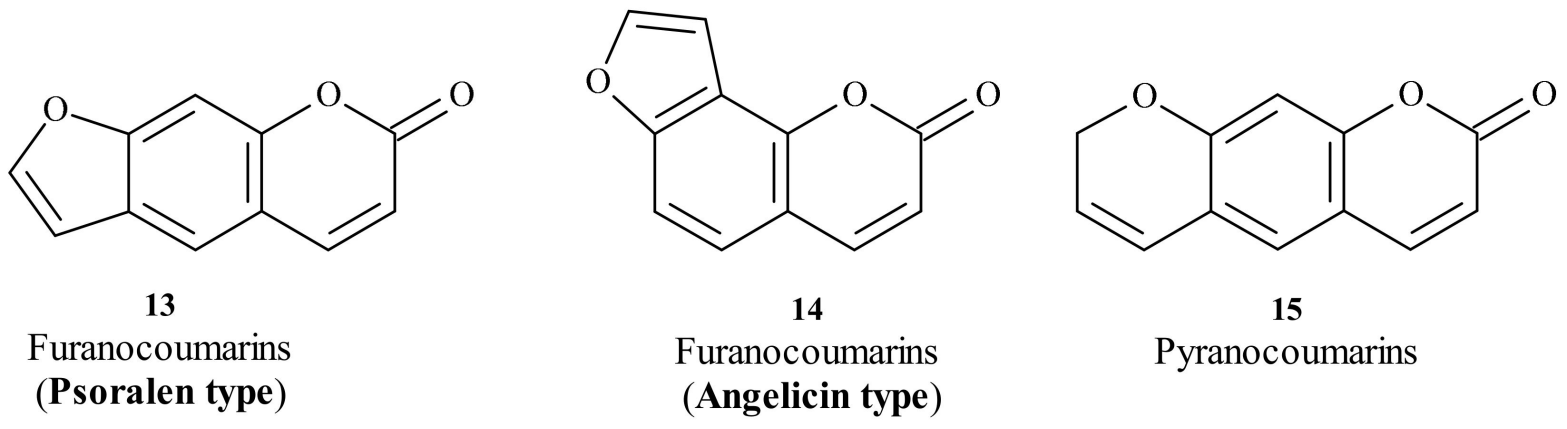
Furanocoumarins and pyranocoumarins.

There are three main subtypes of useful, coumarin-related three-ring compounds presented in Figure 7, i.e., furanocoumarins (e.g., psoralen type, **13** in Figure 7, or angelicin type, **14** in Figure 7), and pyranocoumarins (**15** in Figure 7), but they will not be discussed in this review.

The most popular methods for synthesis of coumarins are Pechmann [37,58] and Knoevenagel condensations [59]. There is also synthetically important intramolecular Wittig reaction [60], which is based on reactive carbonylmethylene(triphenyl) phosphorane intermediates obtained from either salicylaldehydes [61,62] or *o*-quinones [63,64] using phosphorus ylides of general formula Ph_3_P = CH-CO-Lg (Lg = a leaving group, for example, imidazole-1-yl, ethoxyl). Further available methods include, among others, Perkin [65], Reformatsky [66], and Kostanecki–Robinson [67] reactions.

**Scheme 3:**
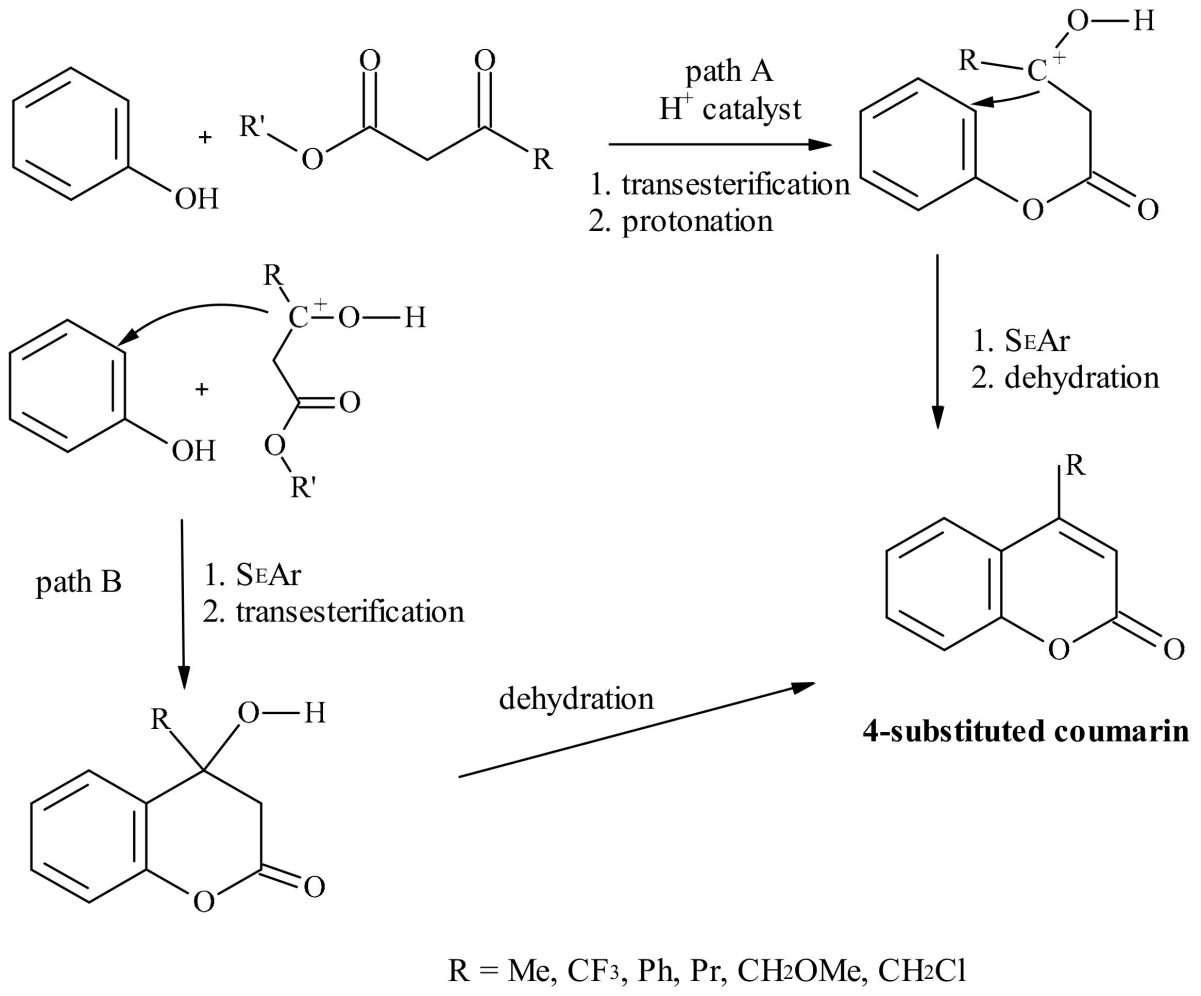
Mechanistic paths that are supposed to operate in Pechmann synthesis of coumarin derivatives.

The most commonly used synthetic route for the preparation of 4-substituted derivatives is Pechmann condensation, which utilizes simple substrates and offers high yields of products. According to this method, the appropriately substituted phenol and *β*-ketoester react in the presence of catalyst (concentrated H_2_SO_4_, trifluoroacetic acid, ZrCl_2_, TiCl_4_, AlCl_3_, etc.). It is assumed in [68] that in the first step the transesterification produces an aryl ester (Scheme 3, path A), in which the *β*-keto group is protonated. The intermediate rapidly cyclizes through an intramolecular condensation (a S_E_Ar type reaction) followed by dehydration. However, there are NMR studies that have concluded that the sequence of events is different, i.e., the condensation begins with the activation of the *β*-keto group (protonation or complexation with a Lewis acid, path B) followed by electrophilic aromatic substitution, cyclization, and final dehydration [69]. It was found that the reaction can be promoted not only by homogenous catalysts, but also by convenient reusable or magnetically recoverable particles [70,71,72] or ionic liquids [73] carrying protic or Lewis acids. Kinetics and mechanistic aspects of their use have been investigated [74]. It was shown that the reaction dramatically speeds up (2 to 10 min instead of 24 h, 60% to 80% yield) if microwave irradiation is applied, even in a household microwave oven [75]. Ultrasonic irradiation also accelerates syntheses and provides the products in good yield [71,76,77]. The properties of many catalysts have been discussed in recently published reviews [78,79].

The Knoevenagel condensation produces 3-substituted coumarins from 2-hydroxybenzaldehydes and 1,3-dicarbonyl compounds. In addition, other compounds containing active methylene group, for example, ROC(O)-CH_2_-X, where X = CN, Cl, Ar, or SO_2_R can be used [80]. This approach allows for having CN, COOH, COOR, or the aryl group at position 3. Typically used basic catalysts include proline, lysine, DBU, and piperidine. Similar to the Pechmann reaction, microwave or ultrasonic irradiation give better results than the temperature driven process (for details see [79]).

**Scheme 4:**
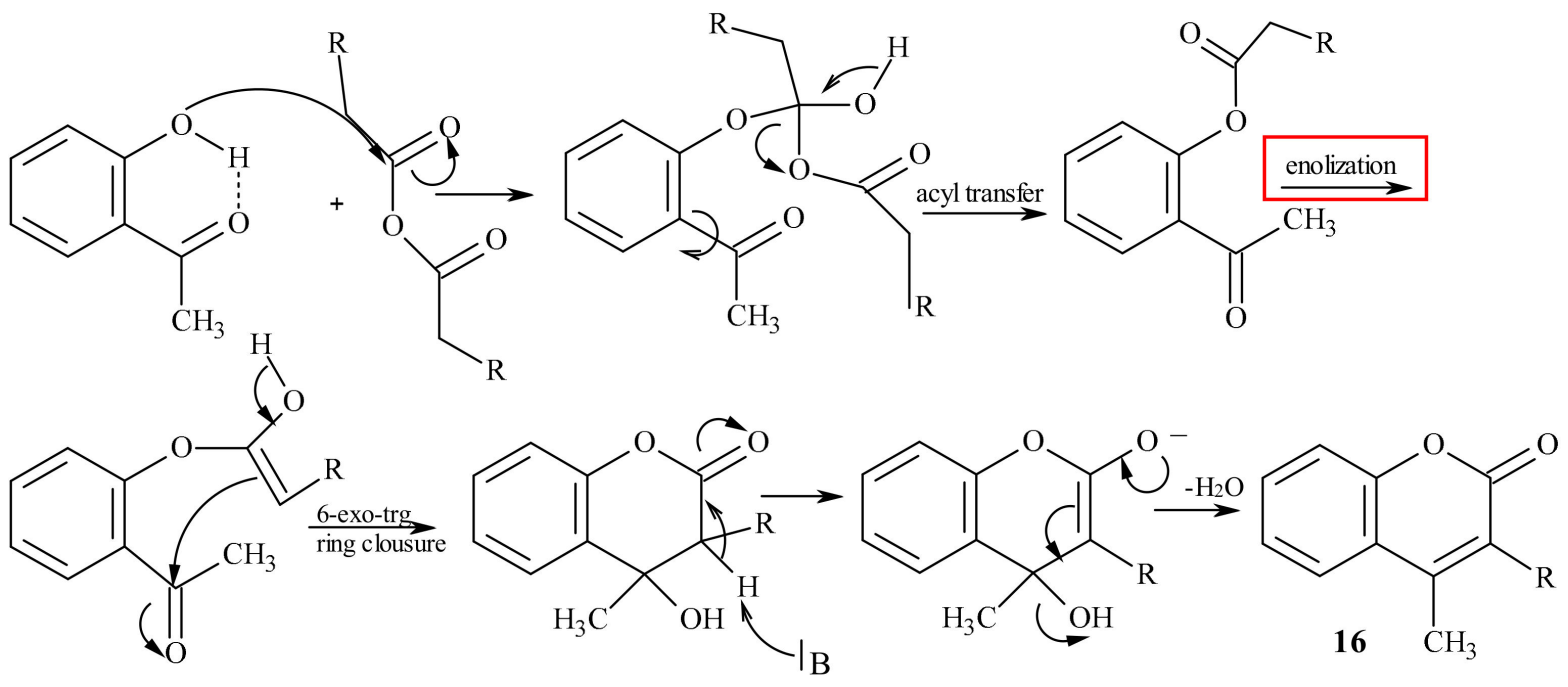
Mechanism of the Kostanecki–Robinson reaction.

3,4-Substituted coumarins (Scheme 4, 16) can been prepared by other methods involving the formation of a 3-4 carbon-carbon bond [81]. For example, the Kostanecki–Robinson reaction of *o*-hydroxyarylalkyl ketones with an acid anhydride and the sodium salt of an acid produces coumarins by the formation of the 3-4 carbon-carbon bond after enolization of the ester (this step is marked by the red rectangular in Scheme 4), but chromones (4*H*-1-benzopyran-4-ones) can be the major products via the ketone enolate, and yields can be variable [82,83,84,85,86].

**Scheme 5:**
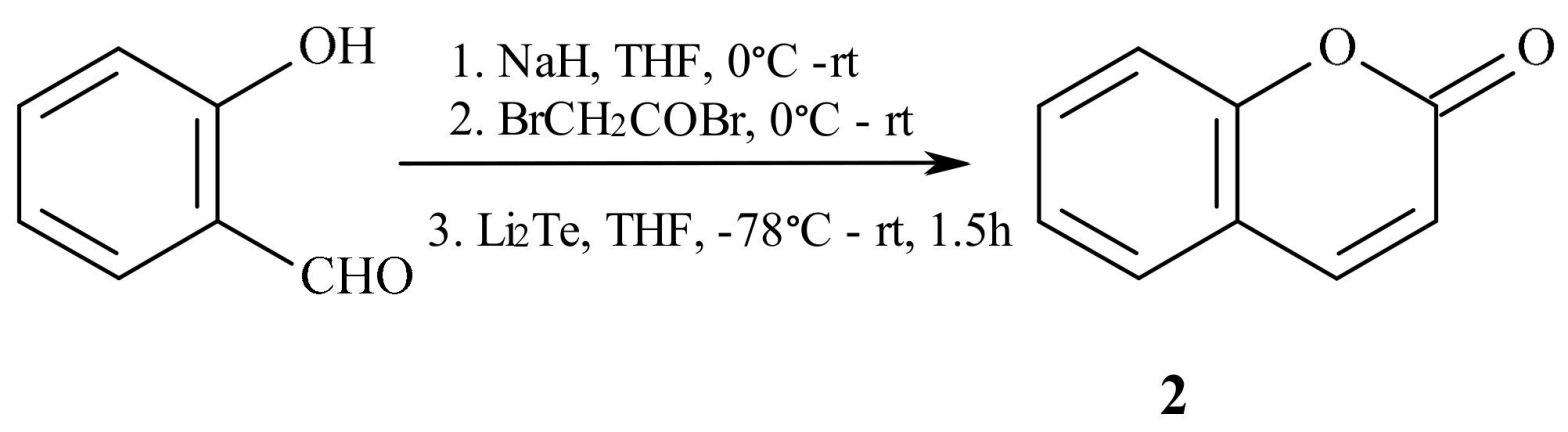
One-pot synthesis of coumarin via the tellurium-triggered cyclization.

A method that avoids the chromone byproducts of the Kostanecki–Robinson reaction is based on the treatment of methyl ester of 2-alkanoyloxybenzoic acids with metallic sodium [87,88] or bases [89,90,91,92]. Coumarins also have been prepared in good yields by a ring-closing metathesis reaction (3-4 bond formation) [93,94] but this reaction is not ideal in respect to the carbon atom economy and requires a ruthenium catalyst. Dittmer and co-workers showed the synthesis of coumarins by the cyclization triggered by lithium telluride (Scheme 5) [95]. Interestingly, sodium telluride is much less effective. 

**Scheme 6:**
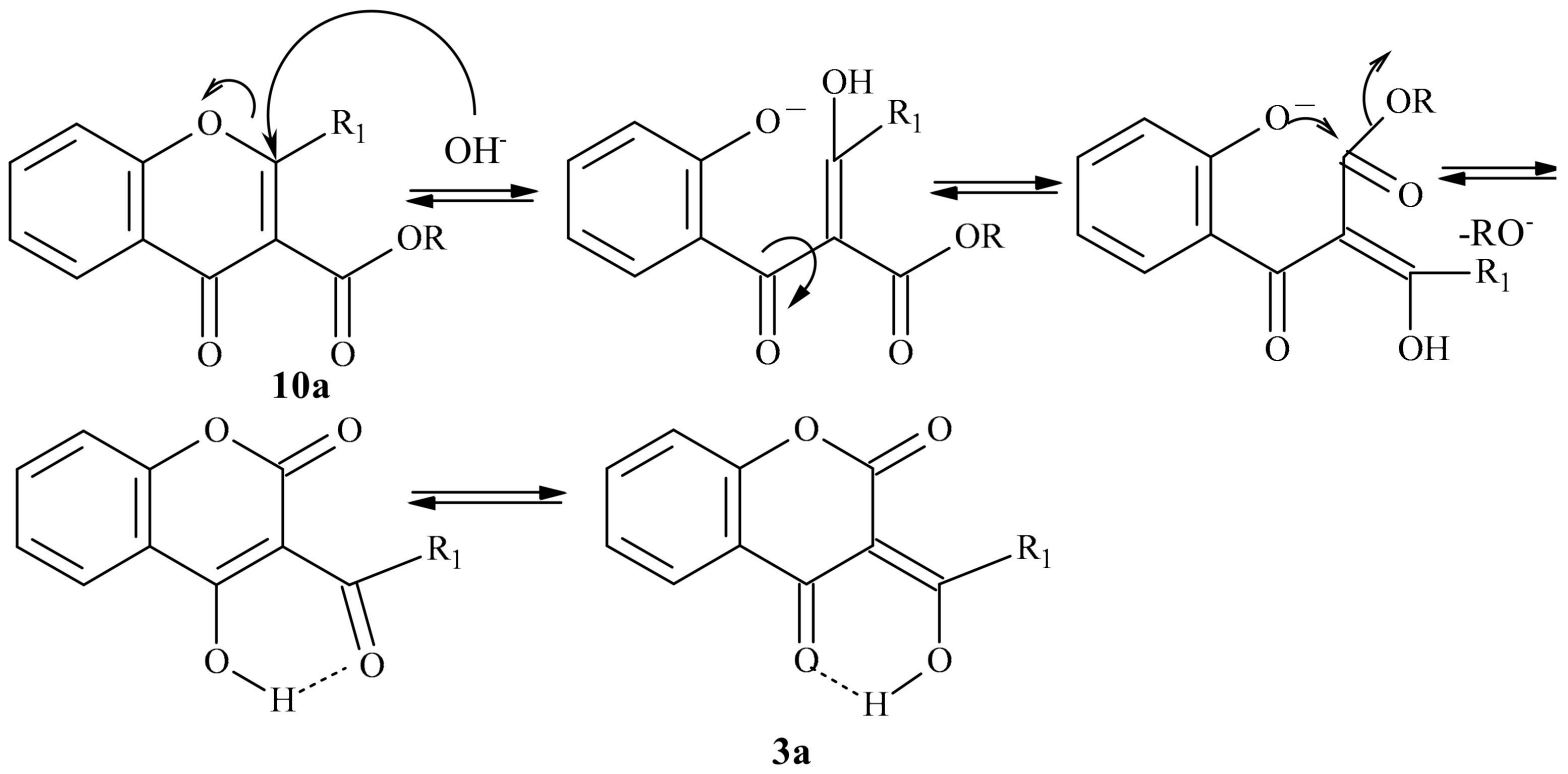
The mechanism of base promoted transformation of a chromone ester derivative into the corresponding coumarin derivative.

In 1974, Klutchko [96] and coworkers showed a convenient and efficient method of synthesis of coumarin derivatives utilizing a hydrolytically promoted ring opening/ring closure reaction of chromone-3-carboxylic esters, with the key rotation step being indicated in Scheme 6 with the contour arrow. In 2017 Abdou published a comprehensive review on methods of synthesis, tautomeric structures and biological properties of 3-acetyl derivatives of 4-hydroxycoumarin (**3a** in Scheme 6) [97].

### 5.1. Warfarin: Stereochemistry and Metabolism

**Scheme 7:**
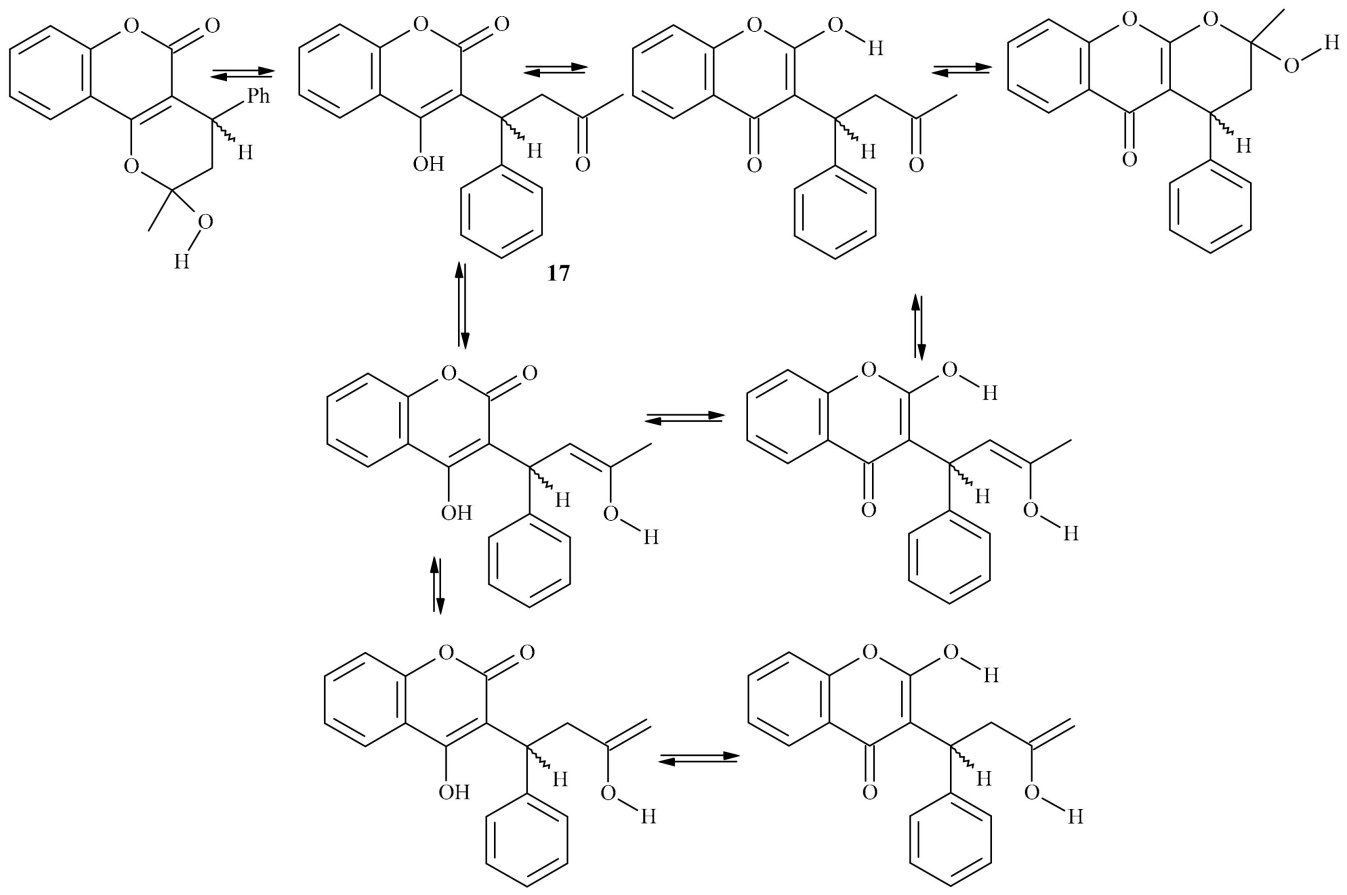
Tautomer substructures of warfarin (**17**), whose combination generates 40 distinct tautomeric forms.

**Scheme 8:**
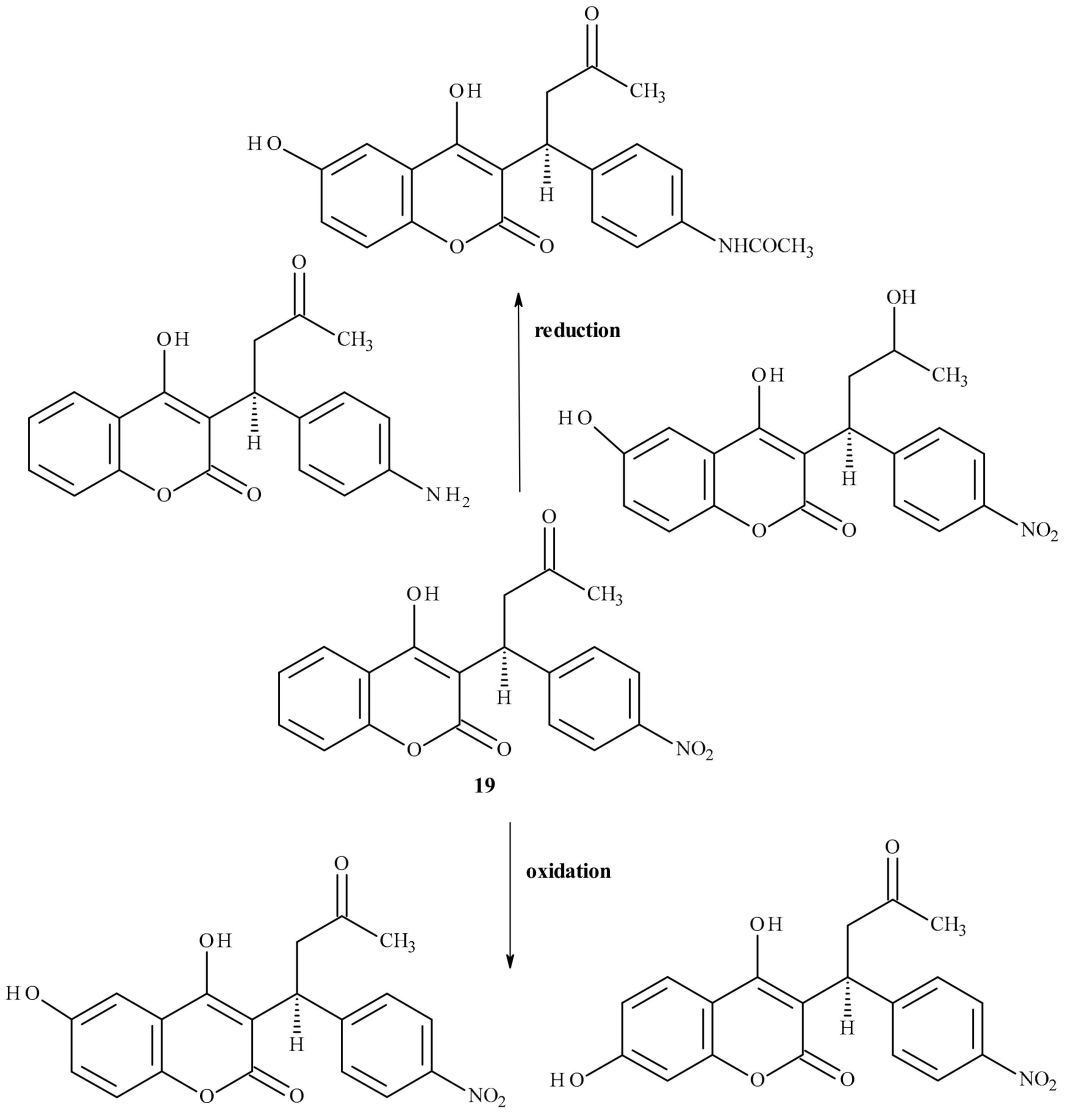
Biotransformation of acenocoumarol in human.

Although warfarin (4-hydroxy-3-(3-oxo-1-phenylbutyl) coumarin (**17** in Scheme 7), 4-hydroxy-3-(3-oxo-1-phenylbutyl)-2*H*-1-benzopyran-2-one) was isolated (from spoiled sweet clover) by Karl Paul Link in 1939, it is still the most often prescribed anticoagulant worldwide [98]. Thus, it deserves a few comments. The lethal dose of warfarin in rats has been found to be between 0.125 and 0.25 mg/kg/day. The drug is administered as a racemate substance, whereas the *S*-warfarin has two to five times greater anticoagulation potency than the *R* counterpart. Warfarin’s maximum concentration in plasma is observed after ca. 90 min and its half-life of 36 to 42 h is usually noted [99]. The enantiomers of warfarin are metabolized mostly in the liver. Primarily, the oxidation reaction by various isoforms of cytochrome P450 takes place, but also reduction reactions can occur in the side chain ketone to yield enantiomeric alcohols. (Scheme 8). In rats, the enantiomers are metabolized by cytochrome p450 monooxygenases CYP 1A1, 2B1, 2C6, 2C11, and 3A2. In humans, *S*-warfarin is metabolized to 7-hydroxywarfarin (a major metabolite) and to 6-hydroxywarfarin (a minor one) by CYP 2C9. The *R*-enantiomer is metabolized to 6-, 8-, or 10-hydroxywarfarin derivatives by CYP 1A2, 2C19, or 3A4, respectively [100]. However, these hydroxylation processes are often affected by other factors, for example, by the coenzyme Q10 [101].

Heteroatoms present in the warfarin molecule allow for the existence of many tautomeric forms. In 2015, Marc C. Nicklaus (Center for Cancer Research, NIH, Frederick, MD) wrote that warfarin “can exist in solution in 40 distinct tautomeric forms through both prototropic tautomerism and ring−chain tautomerism” [102]. However, ^1^H and ^13^C NMR analyses revealed that the drug exists mainly as a 70% *SR’*/28% *SS’* mixture of the cyclic hemiketal diastereomers or their enantiomers *RS’/RR’*, along with a small portion of an open-chain form (Scheme 7).

### 5.2. Analogs of Warfarin

**Figure 8:**
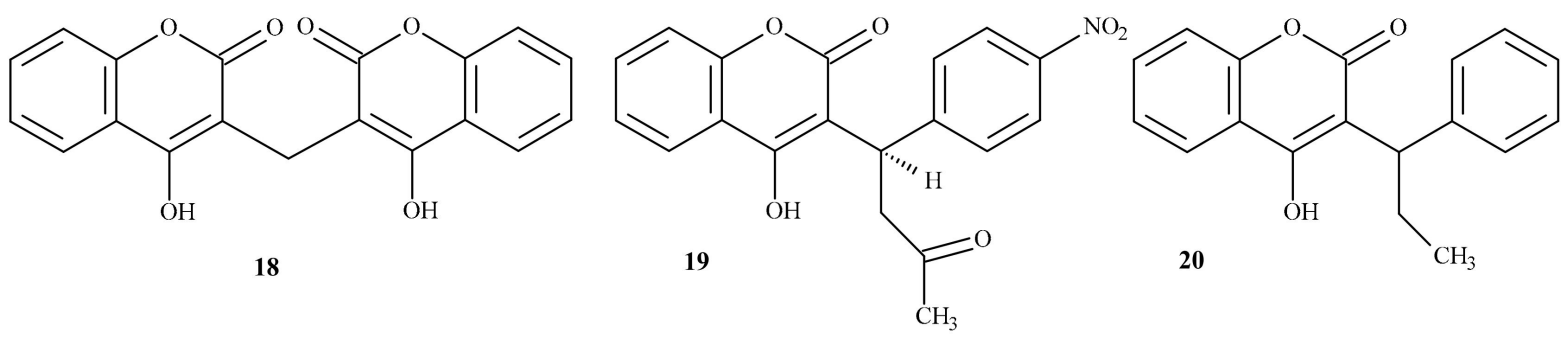
4-Hydroxy-coumarin drugs. (**18**) dicoumarol; (**19**) acenocoumarol; and (**20**) phenprocoumon.

This section begins with dicoumarol (**18** in Figure 8, 3,3′-methylenebis(4-hydroxy-2*H*-chromen-2-one), which barely is a structural analog of warfarin (**17** in Scheme 7). Nonetheless, it is an important predecessor of warfarin, which was employed as an anticoagulant drug until the mid-1950s. Its structure, containing a coumarin motif, was determined by Stahman, in 1941 [88]. Dicoumarol was tested as an anticoagulant agent after the discovery of the cause of sickness and death in cattle. In the 1920s, on the Northern Plains of America, previously healthy cattle began dying of internal bleeding with no obvious reason. Due to the lack of a pathogenic organism or nutritional deficiency as suspects responsible for the bleeding, the diet of the livestock was analyzed. It was, then, found that wet hay became infected by molds such as *Penicillium nigricans* and *Penicillium jensi*, which produced dicoumarol, a metabolite responsible for the disease. In 1945, Karl Link proposed the idea of using a dicoumarol as a rodenticide, but it acted too slowly. After experiments with 150 naturally occurring coumarin derivatives, one compound, later named warfarin, was found to be remarkably active. Initially it was advertised as rodenticide, but after the transition to clinical application in 1955, warfarin was given to President Dwight Eisenhower suffering from a myocardial infarction.

Warfarin exerts high anticoagulation potency and offers high water solubility and good pharmacokinetic profile (good oral bioavailability and efficient absorption from the digestive tract), however, 4-hydroxy-coumarin derivatives (Figure 8), such as acenocoumarol ((*R,S*)-4-hydroxy-3-[1-(4-nitrophenyl)-3-oxobutyl]-2*H*-1-benzopiran-2-one, **19** in Figure 8) or synthetic phenprocoumon (Marcoumar, ((*R*,*S*)-4-hydroxy-3-(1-phenylpropyl)-2*H*-chromen-2-one, **20** in Figure 8) have also been used for the prevention and treatment of thromboembolism [103].

Acenocoumarol acts rapidly and the maximum concentration in plasma is achieved in about 2 h. Whereas an 8 to 11 h half-life time is typically noted, prothrombin time returns to normal 24 to 48 h after the end of the drug intake. In animals and humans, the drug causes no gastrointestinal problems if administered doses are in a normal therapeutic range [104]. Acenocoumarol is metabolized in humans on two paths which involve reduction or oxidation. The reduction converts the nitro group into an amine substituent, or the ketone group into a hydroxyl moiety. Oxidation of acenocoumarol leads to 6- and 7-hydroxy metabolites (Scheme 8).

The anticoagulation effect of phenprocoumon (**20** in Figure 8) is connected with a significantly decreased concentration of factor VII and a less decreased concentration of factor V [105].

**Figure 9:**
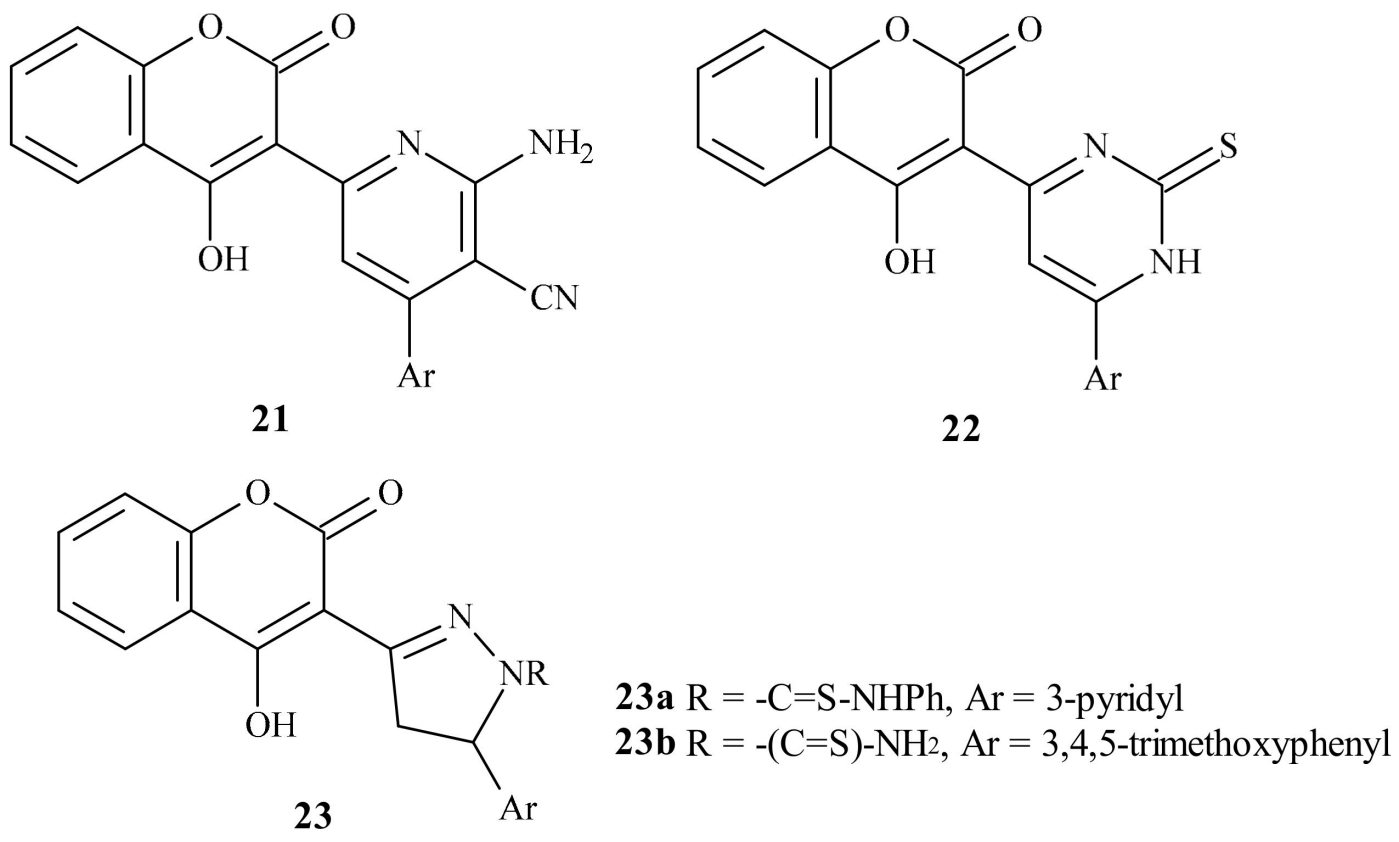
The examples of 4-hydroxycoumarin derivatives, which antagonize VKOR.

Other examples of 4-hydroxycoumarin derivatives which antagonize VKOR are those bearing at the position 3 substituted pyridyl (**21** in Figure 9), pyrimidinyl (**22** in Figure 9), and 3-pyrazolyl (**23** in Figure 9) moieties. Comparative in vivo measurements (clotting time (CT) and prothrombin time (PT)) with warfarin as a reference showed that the pyrazolyl derivative bearing –(C=S)-NHPh attached to the nitrogen atom N1 (**23a** in Figure 9, Ar = 3-pyridyl)) was the most active (19.4%). The pyrazolyl derivative **23b** in Figure 9, with Ar = 3,4,5-trimethoxyphenyl was inactive (see Figure 9) [106].

**Figure 10:**
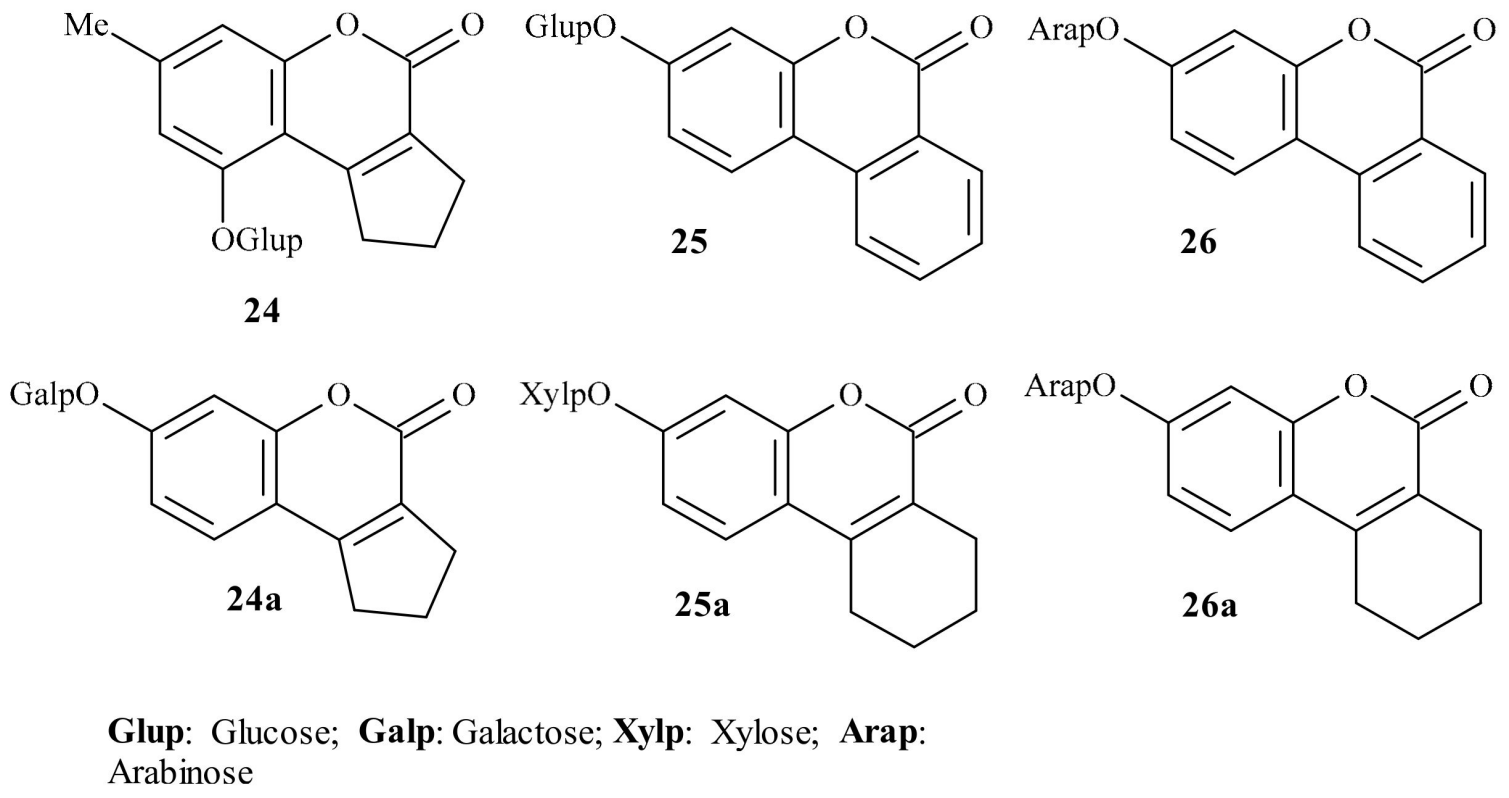
5-Hydroxy- and 7-hydroxy-3,4-cycloannelated coumarin D-glucopyranosides.

Garazd et al. synthesized several 5-hydroxy- and 7-hydroxy-3,4-cycloannelated coumarin D-glucopyranosides (by condensation of potassium salts of hydroxycoumarins and acetobromosugars) [107]. Compared with the parent coumarins, their glycosides (Figure 10) are very soluble in water.

Compound **24** in Figure 10 was only slightly active. Interestingly, compounds **25** and **26a** in Figure 10 over a dose range 0.10–0.30 mg/kg showed hemostatic activity. Compound **24a** in Figure 10 at 0.1 mg/kg showed the anticoagulant activity similar to heparin. Compound **25a** in Figure 10 (at 0.3 mg/kg; structurally similar to hemostatic **26a** in Figure 10, bearing Xyl*p* instead of Ara*p*) and compound **26** in Figure 10 (at 0.25 mg/kg; structurally similar to hemostatic **25** in Figure 10, bearing Ara*p* instead of Glu*p*) were slightly more active than heparin, with CT extended by ca. 6% and 20%, respectively.

**Figure 11:**
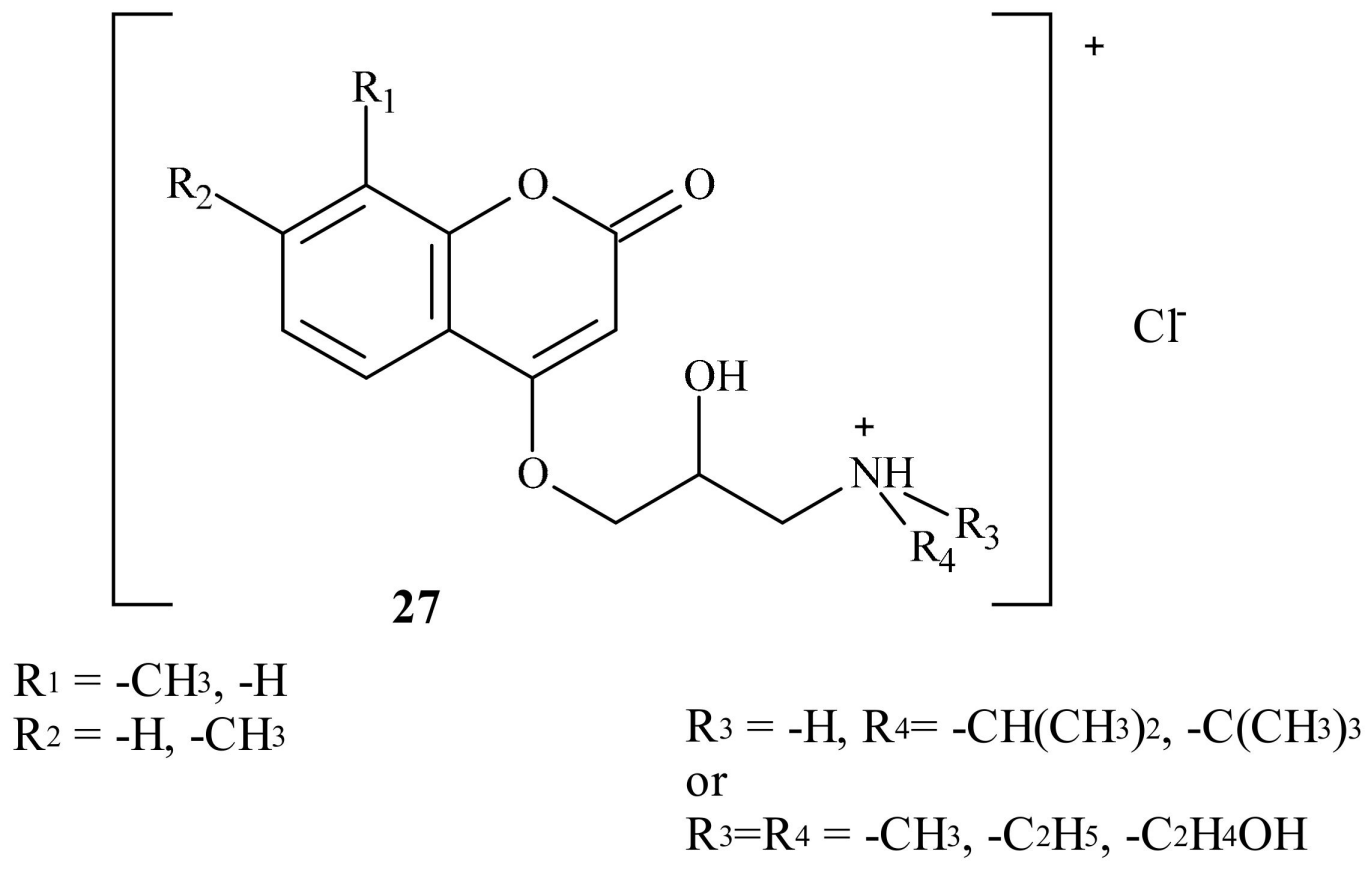
Methylated coumarins with an alkylaminohydroxypropoxy side chain.

The anticoagulant effect accompanied by antihypertensive activity was noted for methylated coumarins bearing an alkylaminohydroxypropoxy side chain at position 4 (**27** in Figure 11). These derivatives caused a significant increase in bleeding and clotting time in rats as compared with the reference warfarin. The mechanism of their dual activity has not been identified [108].

**Scheme 9:**
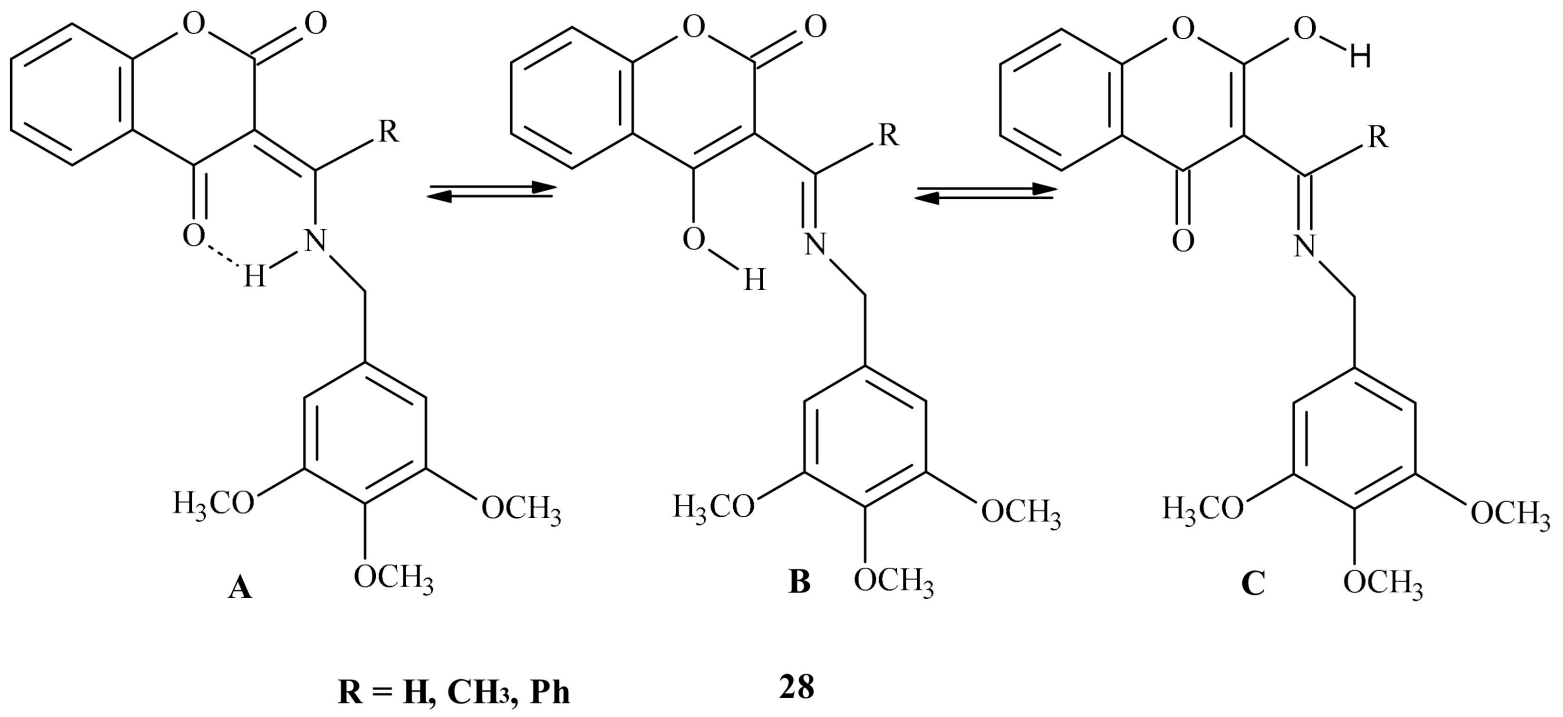
Possible tautomeric forms of **28** bearing the 3,4,5-trimethoxybenzylamine (**28A**) or 3,4,5-trimethoxybenzylimine moiety (**28B** and **28C**).

**Figure 12:**
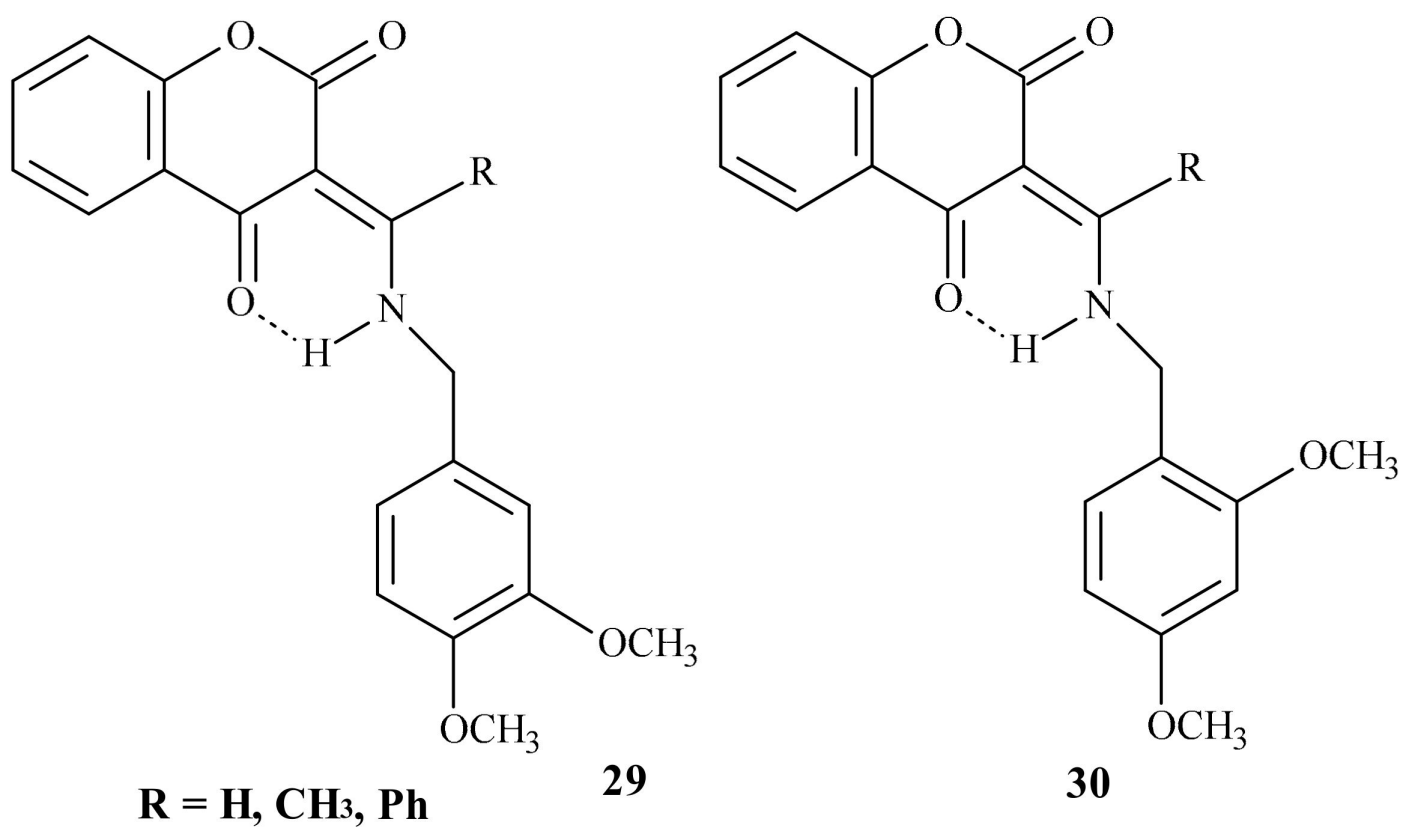
Coumarin derivatives **29**–**30** with two OMe groups in the benzylamine moiety.

Kasperkiewicz and coworkers synthesized new 3-methylidene-4-oxo-coumarin derivatives (**28A** in Scheme 9) which could adopt tautomeric forms (**B** and **C** in Scheme 9), however, X-ray studies revealed the presence of only **A** and **B** structures (Scheme 9), with the ring motifs formed by weak hydrogen bonds [109]. The latter tautomer has hydroxyl and imine functions (Scheme 9) and is somewhat similar to the warfarin molecule. The carbonyl-amine structures of analogs bearing in the C-3 position either 2,4- or 3,4-dimethoxybenzylamine moiety (**29** and **30**, respectively) are shown in Figure 12.

The compounds **28**–**30** (in Scheme 9 and Figure 12) were docked to the vitamin K epoxide reductase complex (subunit 1) and human serum albumin (HSA) to assess their possible anticoagulant effect in comparison to the warfarin enantiomers [109].

**Figure 13:**
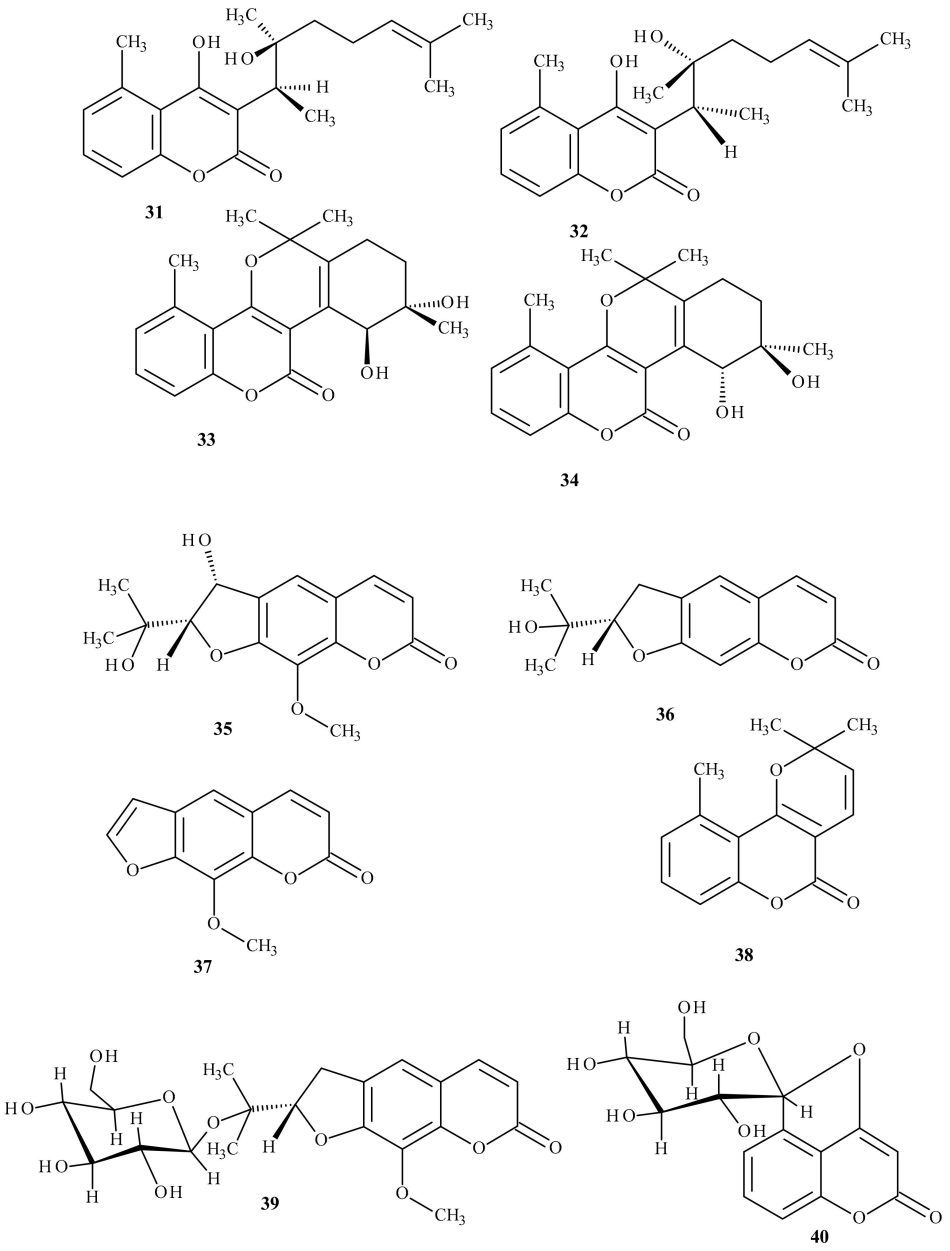
Coumarin related compounds isolated from *Ainsliaea fragrans.*

Concomitantly with synthetic efforts, new coumarin derivatives were isolated from natural sources. Six new 4-hydroxycoumarin derivatives (some with a rare polycyclic pyrano[3-2c]carbon skeleton) and a few psoralene derivatives were isolated from an *Ainsliaea fragrans* herb (**31**–**40**, Figure 13) [110]. Preliminary studies have showed that compound **35** (Figure 13) was a potent anticoagulant with neither hepatic nor renal significant toxicity.

**Figure 14:**
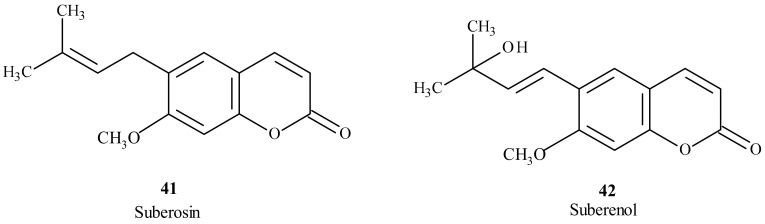
Structures of suberosin (**41**) and suberenol (**42**).

Golfakhrabadi and coworkers isolated two coumarins, suberosin (**41** in Figure 14) and suberenol (**42** in Figure 14), from *Ferulago carduchorum*. These compounds prolonged the prothrombin time at doses of 3 and 6 mg/kg for suberenol, and at dose 6 mg/kg for suberosin [111].

**Figure 15:**
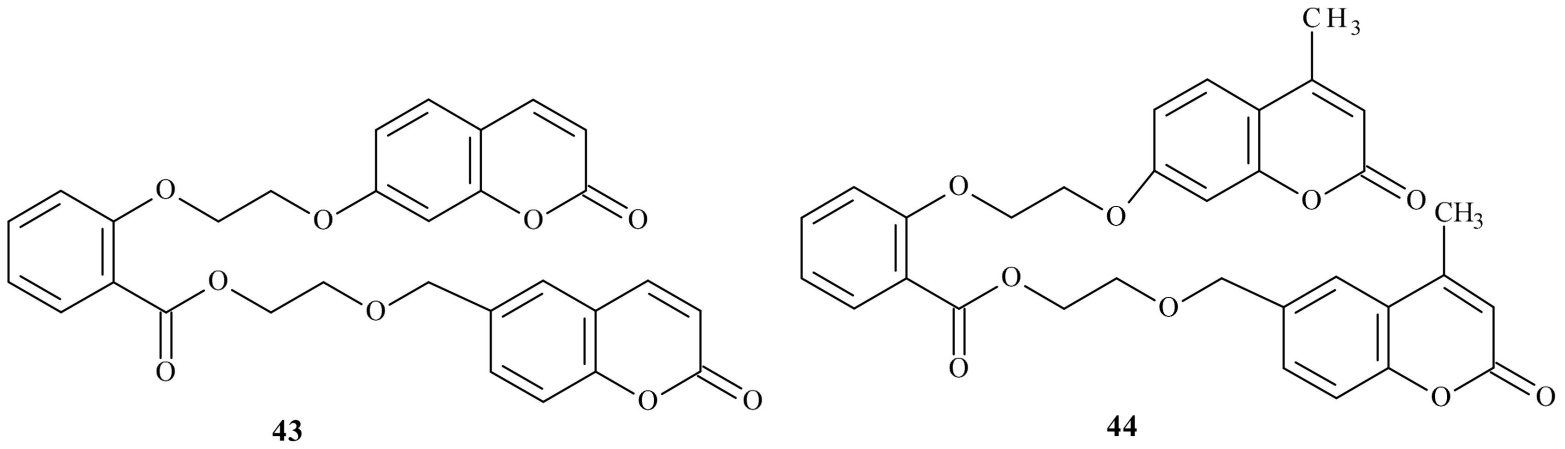
Structure of anticoagulants **43** and **44**.

Bang and coworkers synthesized a series of new conjugates of phenolic acids and coumarin starting from 7-(2-bromoethoxy) coumarin derivatives. For several ether and ester derivatives, the anticoagulant activity was assessed by measurement of the prothrombin time. For the most active two compounds **43** and **44** (Figure 15), PT = 10.88 and 13.10 s were noted, so these compounds are 1.5 times more active than warfarin [112].

**Figure 16:**
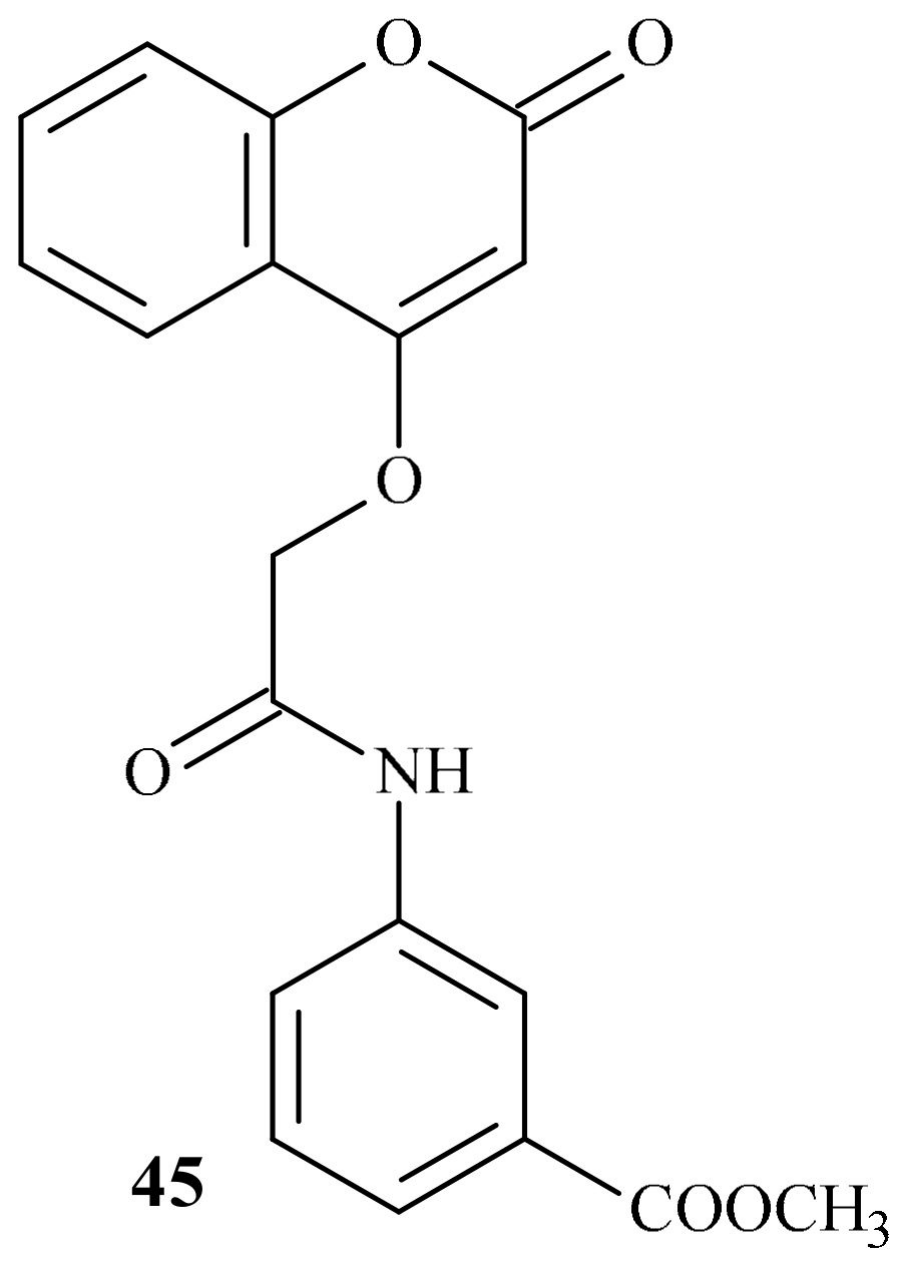
Structure of compound **45.**

Prashantha and coworkers synthesized ten novel coumarins by condensation of 4-hydroxy-coumarin or 7-hydroxy-, 4-methyl-coumarin with acylated amines but none of them showed significant anticoagulant activity and only four compounds were moderately active [113]. The longest clotting time (140 sec) was found for compound **45** (Figure 16), but it is much less than for warfarin (320 s).

Many coumarin derivatives are present in natural extracts. Using three solvents (MeOH, EtOH, and water) and microwave assisted extraction at various temperatures, Channabasava prepared nine partially purified extracts from an Indian plant *Sonchus oleraceus*, in which the presence of coumarin components was confirmed by tests with ferric chloride/nitric acid (FeCl_3_/HNO_3_), and 1N sodium hydroxide (NaOH) solution. The extracts were subjected to APTT and PT anticoagulation assays and strong anticoagulant properties (concentration dependent clotting times for APTT 90 to 220 s and PT 40 to 180 s) were observed for one of the methanolic extracts [114]. However, the active compounds remain unidentified.

**Figure 17:**
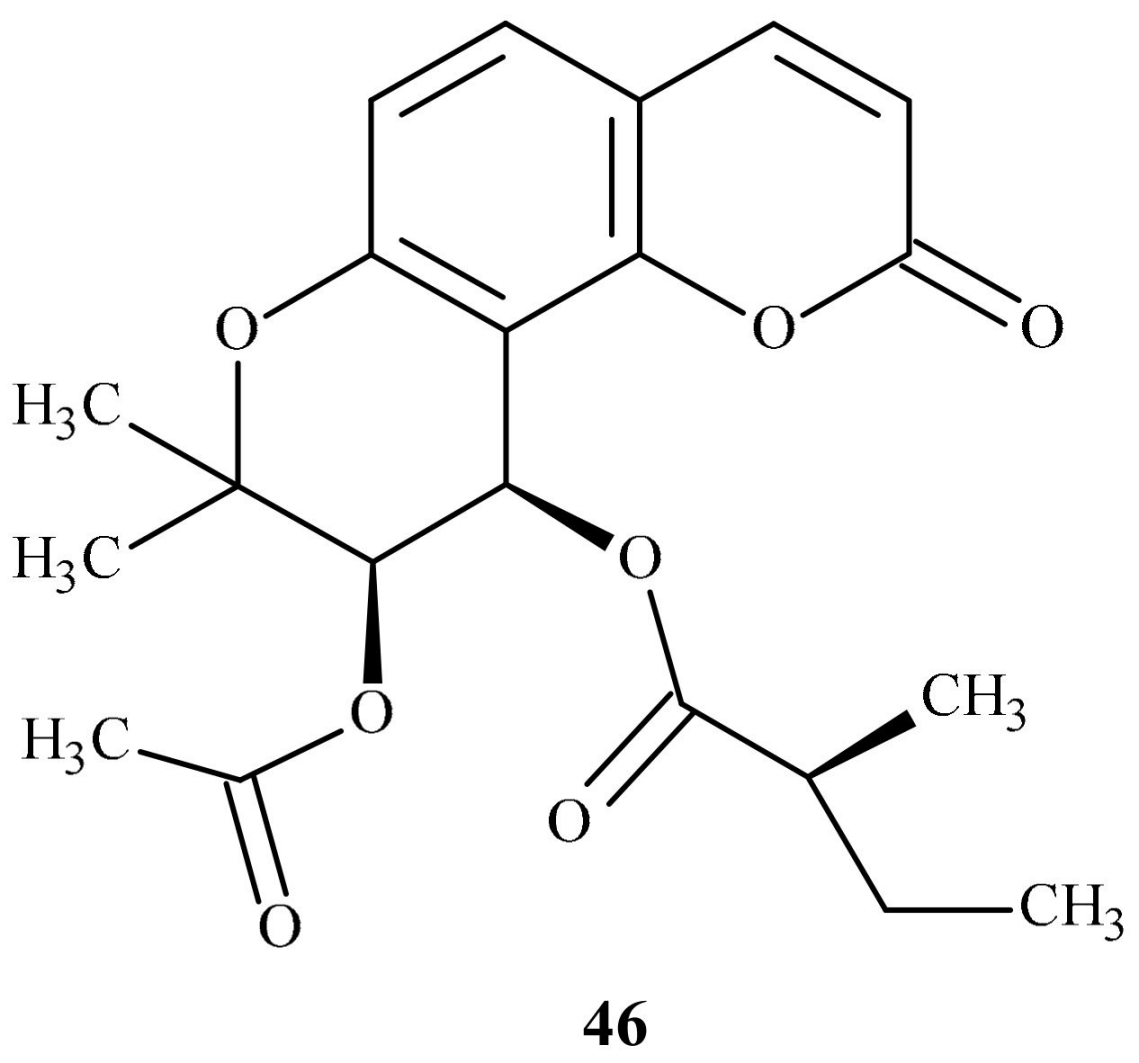
Structure of hyuganin C **46.**

Shimizu and coworkers prepared an ethanolic extract from a Japanese plant *Angelica shikokiana*, which, additionally to the earlier reported anti-inflammatory, antioxidant, and antilipase activities, also had interesting antiplatelet and anticoagulant activities. Three coumarin related compounds, i.e., hyuganin C (Figure 17, **46**), isoepoxypteryxin, and isopterixin were isolated and hyuganin C had a moderate anticoagulant activity as well as a moderate antiplatelet activity against ADP-induced platelet aggregation [115].

An extract obtained from a *Melilotus officinalis* plant used in a volume of 25, 50, and 75 μL showed a dose dependent prothrombin time of treated blood of 46 s, 140 s, and no-clot effect, respectively [116]. The extract was fractionated by column chromatography and anticoagulant activity was assessed for nine fractions, in which several coumarins were identified as: dicoumarol (18 in Figure 8), 2-hydroxy-4-methylene-5-oxo-2,3-dihydrofurane[2-3a][1]benzopyrane, 2-hydroxy-5-methylene-6-oxo-2H,6H,-3,4-dihydropyronal[3,2-C][1]benzopyranone, 2-hydroxy-5-oxo-3,4-dihydrofuran[3-2a][1]benzopyrone, 2-hydroxy-5-oxo-3,4 dihydropyronal[3,2-C][1]benzopyrane, as well as 6-amino-, 6-cyano-, 6-carboxy-, and 4-hydroxycoumarin. However, their overall concentration was relatively low.

In *Artemisia dracunculus* leaf extract (collected from the Sarajevo district) five coumarin derivatives were identified (Table 3) and anticoagulation tests were done, which showed their dose dependent activity [117].

## 6. Summary

In this review, we described more than fifty coumarin derivatives of either natural or synthetic origin. The selection was based on their ability to inhibit a vitamin K epoxide reductase complex, which is a feature beneficial for patients suffering from too strong blood coagulation. Because of very complex biochemical relations governing the processes of clotting and enzymatic removal of clots (briefly presented in Section 2), inhibition of VKOR must be considered a relatively unsafe treatment with a substantial risk of even fatal side effects. Unfortunately, for a number of patients the already developed coumarin-based drugs are ineffective or their efficacy is much reduced because of mutations in the VKOR protein (for details see Section 4). Thus, an intensive search for new compounds is being carried out worldwide. In our review, we tried to compare the activity of new compounds possessing anticoagulant properties with known coumarin drugs, although crude or fractionated extracts from plants, which have been known for a wide spectrum of activity, were often reported. In many instances the active ingredients remain unidentified. The analysis of structures of those already characterized allowed us to conclude that coumarin derivatives bearing a methoxy or hydroxy group in positions C-3 or C-6 are often potent anticoagulants. Unfortunately, there are still no rules linking their structural features with the mechanism(s) of their action.

## Figures and Tables

**Table 1:** Amino acid mutations in VKOR related to CMRES (based on https://www.uniprot.org/uniprot/Q9BQB6) [31].

Position of Amino Acid Residue	Mutation
26	A → T
29	V → L
36	D → G
36	D → Y
45	V → A
52	S → W
56	S → F
58	R → G
59	W → C
59	W → L
66	V → G
66	V → M
71	G → A
77	N → S
77	N → Y
123	I → N
128	L → R

**Table 2:** Amino acid residues vital for the VKOR functioning or strength of the coumarin inhibitory effect (based on https://www.uniprot.org/uniprot/Q9BQB6) [32,33].

Position of Amino Acid Residue	Mutation	Enzyme Activity	Coumarin Inhibitory Effect
16	C → A or S	Reduced by about 80%	
35	R → P	Nearly abolished	
43	C → A or S	Reduced	
51	C → A or S	Reduced	
56	S → P	Nearly abolished	
57	S → A	Nearly abolished	
85	C → A	Reduced by about 25%	
85	C → S	Reduced by about 75%	
96	C → A or S	Reduced by about 70%	
98	R → D or E	Reduced by about 80%.	Decreased
98	R → K	No effect	Decreased
120	L → Q	Decreased moderately.	Decreased
128	L → Q or S	Decreased by about 80%.	Decreased
132	C → S	Nearly abolished	
135	C → S	Nearly abolished	
139	Y → C, G or S	Decreased moderately	Strongly decreased
139	Y → F	No effect	Strongly decreased

**Table 3:** Coumarin derivatives identified in *Artemisia dracunculus.*

Coumarin Derivatives	Structures
7,8-Dihydroxy-6-methoxy-coumarin	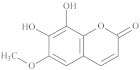
Umbeliferone	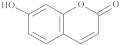
3-Hydroxycoumarin	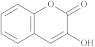
Scopoletin	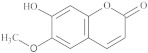
6,7-Dihydroxycoumarin